# Developmental Environmental Chronotoxicity by Polystyrene Nanoplastics Exacerbates High‐Fat Diet‐Induced Pediatric MAFLD Through Circadian Disruption and Mitochondrial Dysfunction

**DOI:** 10.1002/advs.77840

**Published:** 2026-09-27

**Authors:** Peihao Xu, Shuting Huang, Leilei Zhu, Junyi Huang, Kewei Li, Guangming Li, Jiannong Wu, Mingyuan Zhou, Fangmei Zhou, Zhishan Ding, Yingzhi Shen

**Affiliations:** ^1^ School of Medical Technology and Information Engineering The First Affiliated Hospital of Zhejiang Chinese Medical University (Zhejiang Provincial Hospital of Chinese Medicine) Hangzhou Zhejiang China; ^2^ Joint Medical Research Laboratory for Collaborative Prevention and Treatment of Severe Infections with Integrated Chinese and Western Medicine and Translational Medicine Zhejiang Chinese Medical University Hangzhou Zhejiang China

**Keywords:** ampk, biology, clock, fatty liver, mitochondrion, oxidative stress, steatosis

## Abstract

Environmental nanoplastic exposure is increasingly recognized as a contributor to metabolic dysfunction, yet its impact on susceptibility to diet‐induced metabolic dysfunction‐associated fatty liver disease (MAFLD) during development remains unclear. Here, we show that polystyrene nanoplastics (PS‐NPs) exacerbate high‐fat diet (HFD)‐induced hepatic steatosis in an age‐dependent manner. In juvenile, but not adult, mice, PS‐NPs augmented HFD‐driven dyslipidemia, hepatomegaly, and hepatic lipid accumulation without altering energy intake. Mechanistic analyses revealed disruption of the hepatic circadian‐mitochondrial axis, including altered clock gene programs, impaired AMP‐activated protein kinase (AMPK) signaling, mitochondrial dysfunction, and suppressed oxidative metabolism. In hepatocytes, PS‐NPs potentiated free‐fatty‐acid‐induced circadian misalignment, mitochondrial impairment, oxidative stress, and lipid accumulation. AMPK activation by AICAR alleviated steatosis and mitochondrial defects, whereas circadian modulation by SR9009 improved mitochondrial function and hepatocellular injury, highlighting distinct protective mechanisms. Collectively, these findings define early life as a critical window of vulnerability to combined environmental and dietary stressors and introduce the concept of chronotoxicity, the capacity of environmental exposures, such as PS‐NPs, to perturb circadian timing and temporal metabolic organization‐linking nanoplastic exposure to pediatric MAFLD.

## Graphical abstract

Polystyrene nanoplastics exacerbate high‐fat diet‐induced metabolic dysfunction in an age‐dependent manner. Juvenile mice exhibit greater susceptibility, characterized by circadian disruption, impaired mitochondrial function, reduced energy metabolism, and enhanced hepatic lipid accumulation. Pharmacological modulation with AICAR or SR9009 partially restores metabolic homeostasis, highlighting interconnected circadian and mitochondrial mechanisms in pediatric MAFLD.

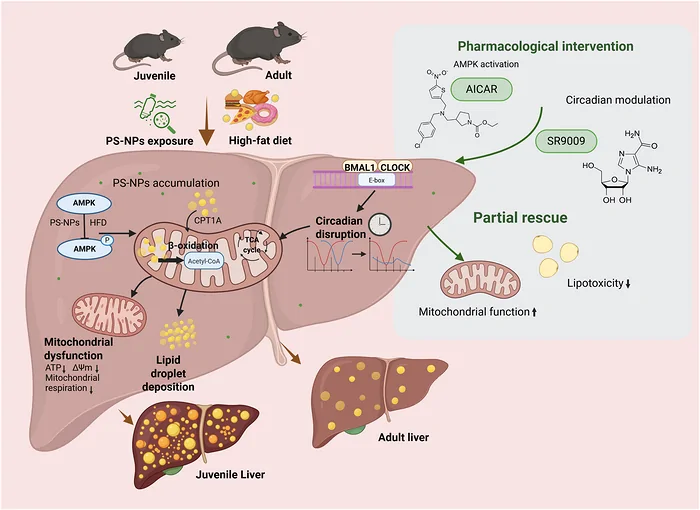

## 1 Introduction

Nanoplastics (NPs) are plastic particles with a size <1 µm. Their small size facilitates biological translocation and systemic distribution, allowing accumulation in organs such as the liver, and they have become a common environmental contaminant [1, 2, 3, 4, 5]. Among various types of NPs, polystyrene nanoplastics (PS‐NPs) are particularly prevalent because polystyrene is extensively used in packaging materials and consumer products and exhibits high environmental persistence [6]. Experimental studies have demonstrated that PS‐NPs exposure induces oxidative stress and disrupts multiple cellular processes, including mitochondrial function, lipid metabolism, and inflammatory regulation. These alterations may compromise metabolic homeostasis and increase susceptibility to metabolic disorders [7, 8, 9]. However, environmental contaminants are rarely encountered independently, and the mechanisms by which PS‐NPs interact with dietary challenges to influence metabolic disease progression remain largely unclear.

Metabolic dysfunction‐associated fatty liver disease (MAFLD) represents a growing global health burden, with a rapid increase in prevalence among children and adolescents [10, 11]. The developmental origins of health and disease (DOHaD) paradigm highlights that environmental insults during critical developmental windows may induce more profound and persistent metabolic consequences than similar exposures occurring in adulthood [12, 13]. During early life, the liver undergoes extensive maturation of mitochondrial bioenergetics, circadian regulatory systems, and nutrient‐sensing pathways, potentially resulting in increased vulnerability to environmental perturbations [14]. Therefore, early‐life exposure to emerging contaminants such as PS‐NPs may represent an overlooked environmental determinant contributing to pediatric metabolic susceptibility.

Recent studies have revealed that PS‐NPs can promote hepatic steatosis through multiple mechanisms, including impaired autophagic degradation of nuclear receptor corepressor 1 (NCoR1), suppression of peroxisome proliferator‐activated receptor α (PPARα)‐mediated fatty acid oxidation (FAO), lysosomal dysfunction, and disrupted lipid catabolism [15, 16]. These findings suggest that PS‐NPs are not merely passive toxicants but active regulators of hepatic metabolic homeostasis. Nevertheless, two critical questions remain unresolved: whether developmental stage determines susceptibility to PS‐NPs‐induced metabolic disturbances under dietary stress, and how PS‐NPs are integrated into hepatic regulatory networks to drive MAFLD progression.

The circadian clock and cellular energy‐sensing pathways are fundamental regulators of hepatic metabolic homeostasis. The molecular circadian system coordinates rhythmic processes involved in lipid synthesis, FAO, mitochondrial function, and energy metabolism, thereby maintaining metabolic flexibility in response to feeding‐fasting cycles [17, 18, 19, 20, 21]. AMP‐activated protein kinase (AMPK), a central cellular energy sensor, interacts with circadian regulatory mechanisms to couple nutrient availability with mitochondrial bioenergetics and substrate utilization. Disruption of circadian regulation, mitochondrial function, or AMPK signaling can impair metabolic adaptation, promote lipid accumulation, and accelerate hepatic steatosis [22, 23, 24, 25]. Although mitochondrial dysfunction has been increasingly recognized as a key consequence of nanoplastic exposure, whether PS‐NPs disrupt the coordinated regulation of circadian rhythms, mitochondrial metabolism, and AMPK signaling to promote MAFLD remains poorly understood.

Here, we investigated the developmental stage‐dependent effects of chronic PS‐NPs exposure on high‐fat diet (HFD)‐induced MAFLD in juvenile and adult mice, complemented by mechanistic studies in hepatocyte models. We demonstrate that early‐life exposure confers heightened susceptibility to PS‐NPs‐enhanced hepatic steatosis and metabolic dysfunction. Mechanistically, PS‐NPs disrupt hepatic circadian rhythmicity, impair mitochondrial bioenergetic capacity, suppress AMPK activation, and reduce fatty acid oxidative metabolism, thereby aggravating lipid accumulation and metabolic stress. Collectively, our findings identify a developmental window of increased vulnerability to combined environmental and dietary challenges and highlight environmental chronotoxicity‐the disruption of circadian timing and temporal metabolic organization by environmental stressors‐as a potential mechanism linking nanoplastic exposure to MAFLD progression.

## 2 Results

### 2.1 PS‐NPs Exacerbate HFD‐Induced Hepatic Steatosis in a Developmental Stage‐Dependent Manner

**FIGURE 1:**
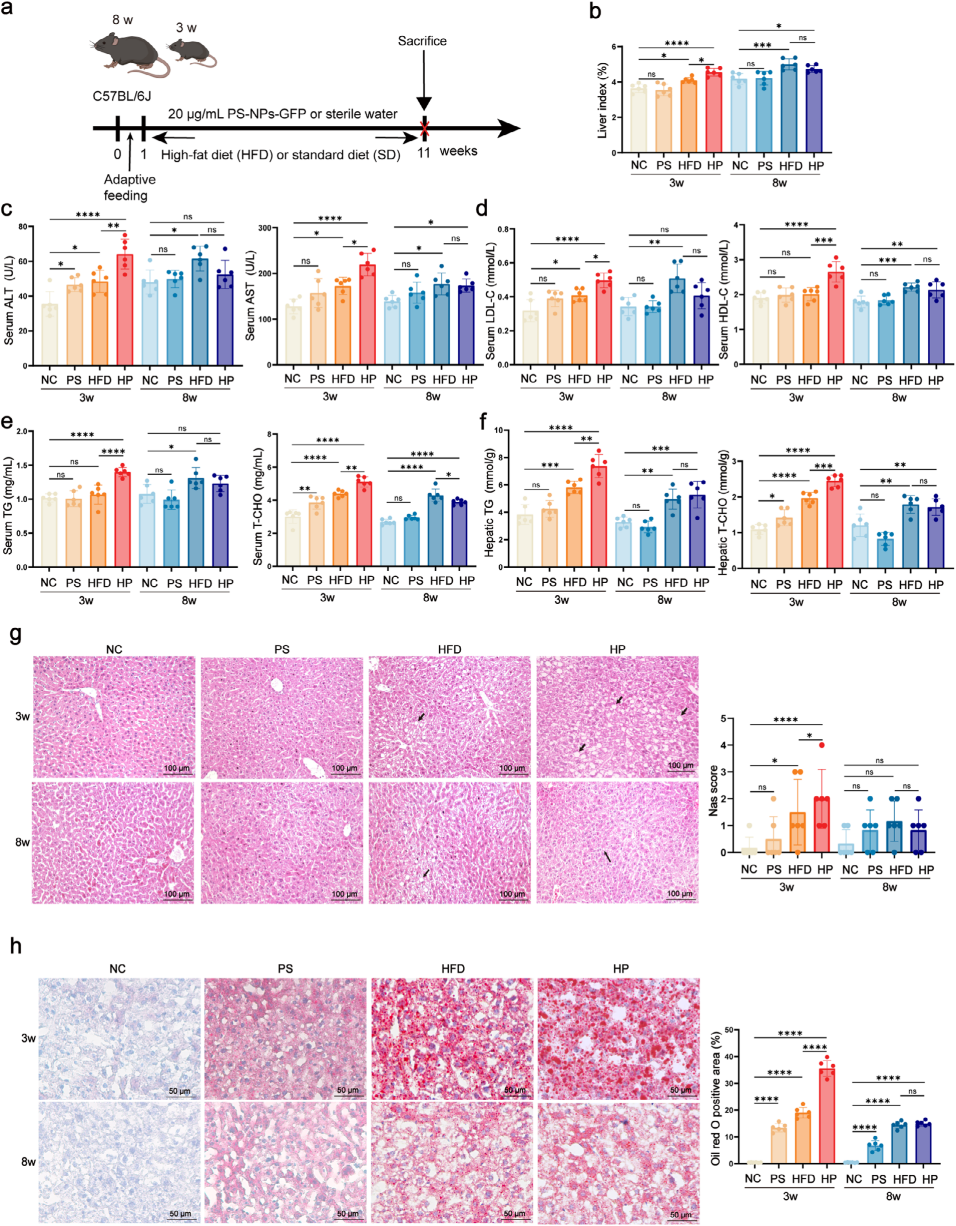
PS‐NPs selectively exacerbate HFD‐induced hepatic steatosis in juvenile mice. (a) Overview of the 10‐week exposure protocol. Male C57BL/6J mice entered the study at 3 or 8 weeks of age. Each age cohort received normal chow or HFD (60% kcal from fat), with or without GFP‐labeled PS‐NPs (100 nm, 20 µg/mL) in drinking water. (b) Liver index (liver weight/body weight × 100%). (c) Serum levels of alanine aminotransferase (ALT) and aspartate aminotransferase (AST). (d) Serum concentrations of LDL‐C and HDL‐C. (e) Serum TG and T‐CHO concentrations. (f) Hepatic TG and T‐CHO contents. (g) Representative hematoxylin and eosin (H&E)‐stained liver sections and corresponding NAFLD activity scores (NAS). Scale bar, 100 µm. (h) Representative Oil Red O‐stained liver sections and quantification of lipid‐positive areas. Scale bar, 50 µm. Data are shown as mean ± SD (n = 6/group). Two‐way ANOVA (HFD × PS‐NPs) with Tukey correction was used. *p < 0.05, **p < 0.01, ***p < 0.001, **p < 0.0001; ns, not significant. NC, normal chow; PS, PS‐NPs; HFD, high‐fat diet; HP, HFD + PS‐NPs.

To determine whether developmental stage influences susceptibility to PS‐NPs‐induced metabolic disturbances, male C57BL/6J mice from two age groups were enrolled, including 3‐week‐old juvenile mice and 8‐week‐old adult mice at the beginning of exposure. Within each age cohort, mice were subjected to a 2 × 2 factorial experimental design combining dietary intervention (standard chow or HFD, providing 60% of calories from fat) and PS‐NPs exposure (0 or 20 µg/mL in drinking water; 100‐nm GFP‐labeled particles) for 10 weeks (Figure 1a).

Throughout the experimental period, body weight‐normalized food and water consumption showed no significant differences among groups (Figure S1a,b). Because animals were maintained under group‐housing conditions, individual PS‐NPs intake could not be directly quantified. Therefore, PS‐NPs exposure levels were estimated based on cage‐level water consumption, PS‐NPs concentration in drinking water, the number of animals per cage, and individual body weight. During the first eight weeks of exposure, the calculated mean weekly PS‐NPs intake was 25.53, 21.69, 24.17, and 24.81 µg/g body weight/week in juvenile PS‐NPs, juvenile HFD + PS‐NPs (HP), adult PS‐NPs, and adult HP groups, respectively. The corresponding cumulative exposure levels were 204.24, 173.56, 193.33, and 198.46 µg/g body weight, respectively (Table S1). HFD feeding significantly increased energy intake compared with chow‐fed controls, whereas PS‐NPs exposure did not further affect caloric consumption in either age group (Figure S1c).

The stability and physicochemical characteristics of the GFP‐labeled PS‐NPs were subsequently evaluated. Scanning electron microscopy (SEM) demonstrated that PS‐NPs remained uniformly dispersed in aqueous solution over three days without obvious aggregation or precipitation (Figure S2a). Dynamic light scattering (DLS) analysis revealed an average hydrodynamic diameter of 115.4 ± 1.5 nm and a low polydispersity index (PDI) of 0.041 ± 0.040, confirming a narrow particle size distribution and stable dispersion. The zeta potential was −30.9 ± 0.7 mV, indicating a negatively charged surface and favorable colloidal stability under the experimental conditions (Figure S2b). Furthermore, comparable hepatic GFP fluorescence signals were detected between juvenile and adult mice, suggesting similar hepatic accumulation of PS‐NPs across different age groups and treatment conditions (Figure S2c).

We next assessed the effects of PS‐NPs on HFD‐induced metabolic phenotypes. PS‐NPs exposure alone did not significantly influence the liver index (liver weight/body weight × 100%) in either juvenile or adult mice. Although HFD markedly increased liver index in both age groups, indicative of hepatic enlargement, additional PS‐NPs exposure further increased liver index only in juvenile mice (Figure 1b). Consistent with these findings, serum ALT and AST levels were significantly elevated in juvenile HP mice compared with mice receiving HFD alone, whereas PS‐NPs did not further increase hepatic injury markers in adult mice (Figure 1c). In contrast, HFD‐induced increases in body weight gain and adiposity were comparable between juvenile and adult mice and were unaffected by PS‐NPs exposure (Figure S3a,b). Similarly, PS‐NPs did not further aggravate HFD‐induced glucose intolerance or significantly alter insulin sensitivity (Figure S3c,d), indicating that the enhanced hepatic injury observed in juvenile HP mice was not attributable to increased obesity or systemic glucose dysregulation.

Serum biochemical analysis further revealed age‐dependent differences in lipid metabolic responses. In juvenile mice, PS‐NPs co‐exposure significantly amplified HFD‐induced elevations in low‐density lipoprotein cholesterol (LDL‐C), high‐density lipoprotein cholesterol (HDL‐C), triglycerides (TG), and total cholesterol (T‐CHO), whereas these alterations were not further affected by PS‐NPs in adult mice (Figure 1d–f). Consistently, histopathological examination demonstrated that juvenile HP mice displayed more severe hepatic lipid accumulation, characterized by increased lipid droplet deposition, hepatocellular ballooning, and extensive vacuolar degeneration compared with HFD‐fed controls. In contrast, PS‐NPs co‐exposure produced limited additional pathological changes in adult mice (Figure 1g,h).

Collectively, these findings demonstrate that chronic PS‐NPs exposure selectively aggravates HFD‐induced hepatic steatosis in juvenile mice despite comparable PS‐NPs exposure levels, hepatic accumulation, and energy intake between age groups. These results reveal an increased developmental susceptibility to nanoplastic‐induced metabolic disruption during early life.

### 2.2 PS‐NPs Amplify HFD‐Induced Circadian and Mitochondrial Transcriptional Alterations in Juvenile Mice

**FIGURE 2:**
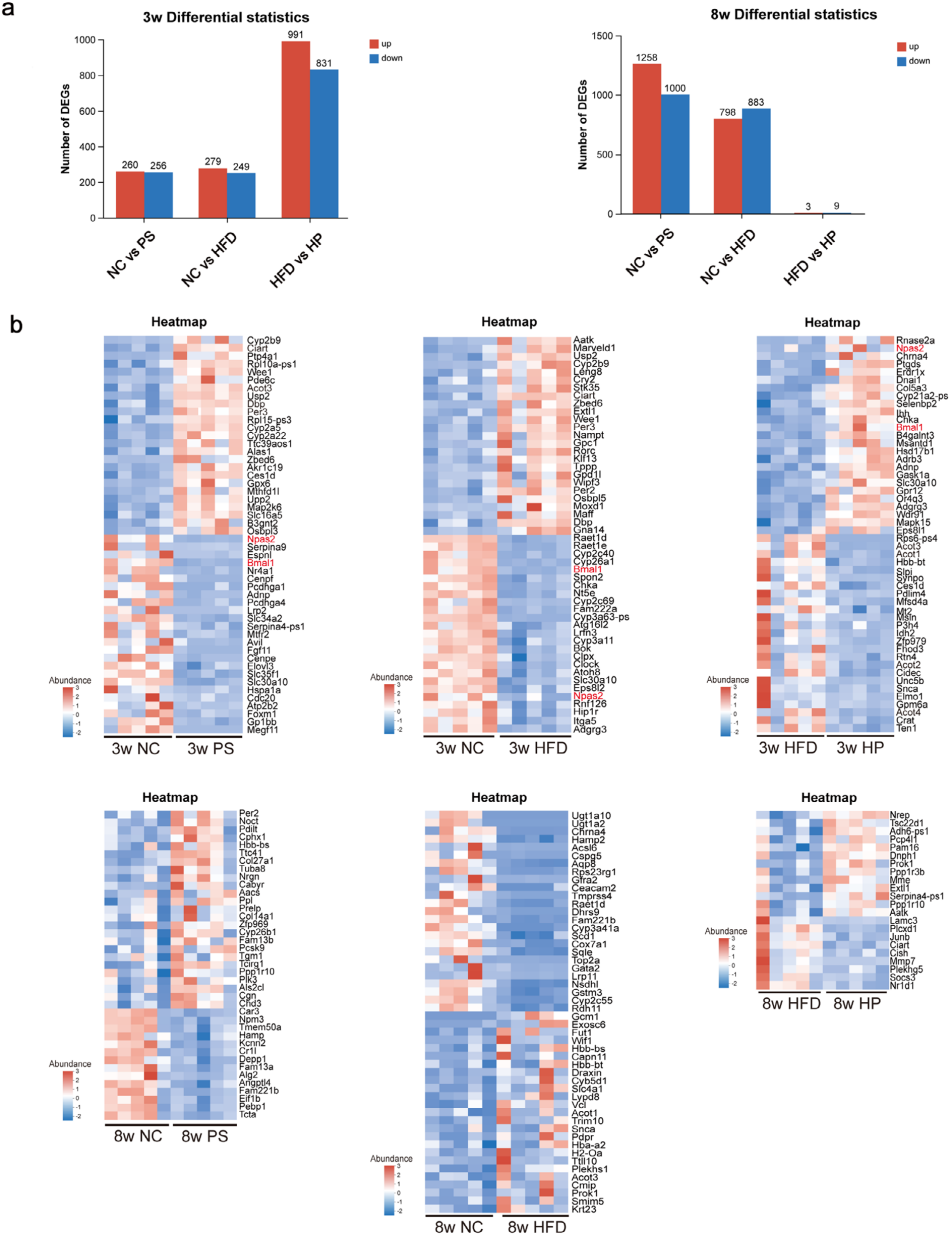
PS‐NPs intensify HFD‐induced transcriptomic alterations and circadian gene dysregulation in juvenile mice. (a) Overview of hepatic transcriptomic profiling in juvenile and adult mice subjected to PS‐NPs exposure, HFD feeding, or combined HFD and PS‐NPs treatment. (b) Heatmaps showing the 25 most significantly upregulated and 25 most significantly downregulated differentially expressed genes (DEGs) with the greatest fold‐change alterations in each indicated comparison. DEGs were defined by DESeq2 using |log_2_ fold change| ≥ 1 and adjusted *p* < 0.05. (n = 5/group). NC, normal chow; PS, PS‐NPs; HFD, high‐fat diet; HP, HFD + PS‐NPs.

RNA‐seq analysis revealed pronounced age‐dependent differences in hepatic transcriptional responses to PS‐NPs exposure. In juvenile mice, PS‐NPs or HFD alone induced moderate transcriptional changes, whereas co‐exposure elicited substantially enhanced gene expression remodeling, indicating that PS‐NPs potentiated HFD‐associated transcriptomic alterations (Figure 2a). In contrast, adult mice exhibited markedly attenuated transcriptional responses, with co‐exposure inducing only limited additional changes, suggesting reduced sensitivity to nanoplastic‐diet interactions.

Among core circadian regulators, *Bmal1* and *Npas2* displayed non‐linear expression patterns in juveniles: both genes were downregulated by either PS‐NPs or HFD alone but partially restored under co‐exposure relative to HFD alone (Figure 2b). Given that profiling was performed at a single circadian time point, these patterns likely reflect feedback regulation or phase‐dependent transcriptional variability rather than restoration of circadian homeostasis.

**FIGURE 3:**
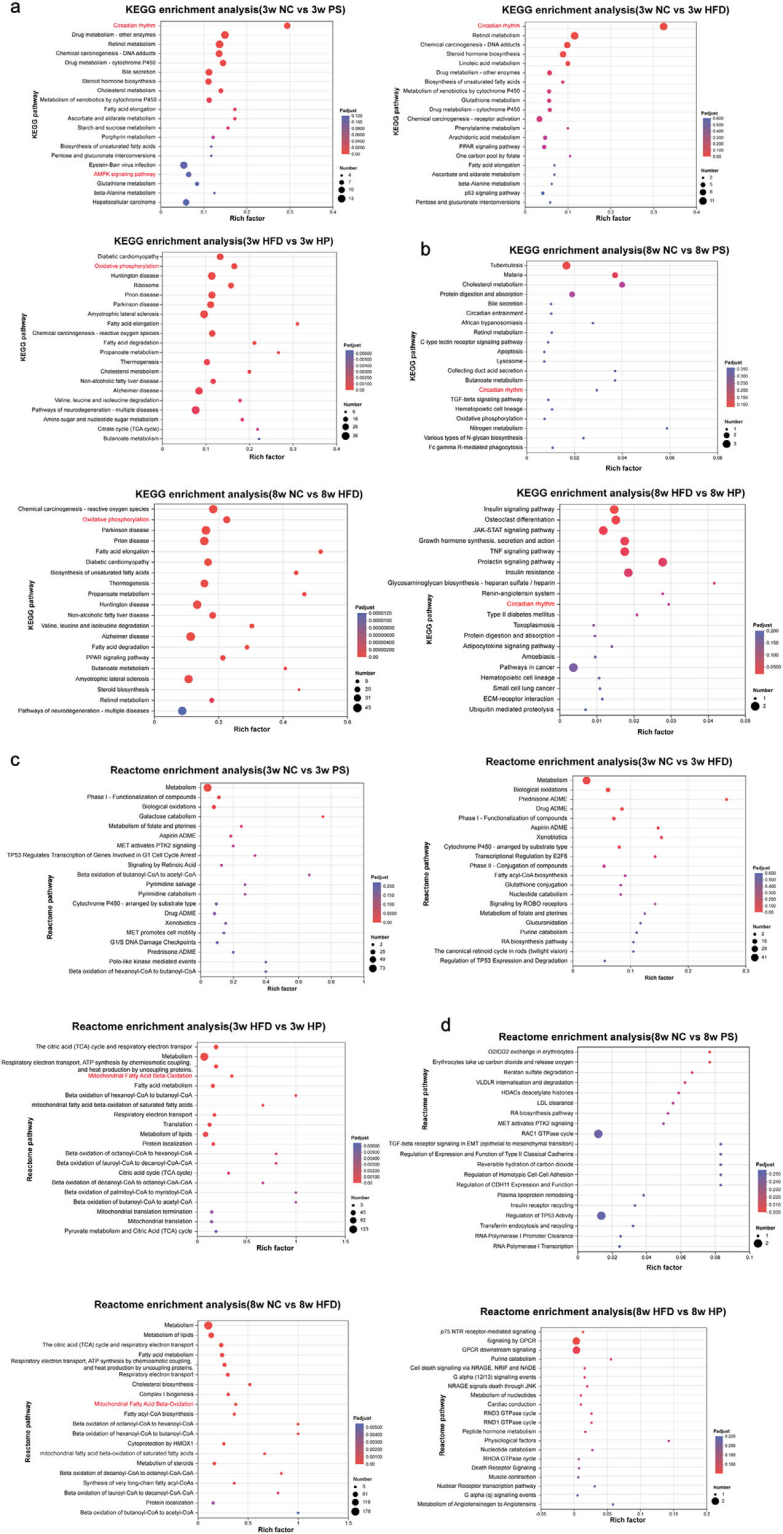
PS‐NPs enhance HFD‐associated circadian and mitochondrial pathway remodeling in an age‐dependent manner. (a) KEGG pathway enrichment analysis of DEGs identified in juvenile mice. (b) KEGG pathway enrichment analysis of DEGs identified in adult mice. (c) Reactome pathway enrichment analysis of DEGs identified in juvenile mice. (d) Reactome pathway enrichment analysis of DEGs identified in adult mice. (n = 5/group) NC, normal chow; PS, PS‐NPs exposure; HFD, high‐fat diet; HP, combined HFD and PS‐NPs exposure.

Pathway enrichment analyses further demonstrated age‐dependent metabolic remodeling. In juveniles, Kyoto Encyclopedia of Genes and Genomes (KEGG) analysis indicated that PS‐NPs or HFD alone primarily affected circadian rhythm and AMPK signaling pathways, whereas co‐exposure preferentially enriched oxidative phosphorylation and other metabolic pathways compared with HFD alone (Figure 3a). In adults, HFD predominantly enriched oxidative phosphorylation and lipid metabolism pathways, while PS‐NPs co‐exposure induced only marginal additional pathway alterations (Figure 3b). Consistently, Reactome analysis revealed that juvenile co‐exposure was associated with significant enrichment of lipid metabolism, mitochondrial fatty acid β‐oxidation, and respiratory electron transport chain pathways (Figure 3c), whereas such mitochondrial metabolic remodeling was substantially less pronounced in adults (Figure 3d).

Overall, these findings indicate that juvenile livers are transcriptionally hypersensitive to combined dietary and nanoplastic stress, with PS‐NPs amplifying HFD‐induced circadian disruption and mitochondrial metabolic reprogramming in an age‐dependent manner.

### 2.3 Combined PS‐NPs and HFD Exposure Disrupts Circadian Regulation and Mitochondrial Metabolic Homeostasis in the Juvenile Liver

**FIGURE 4:**
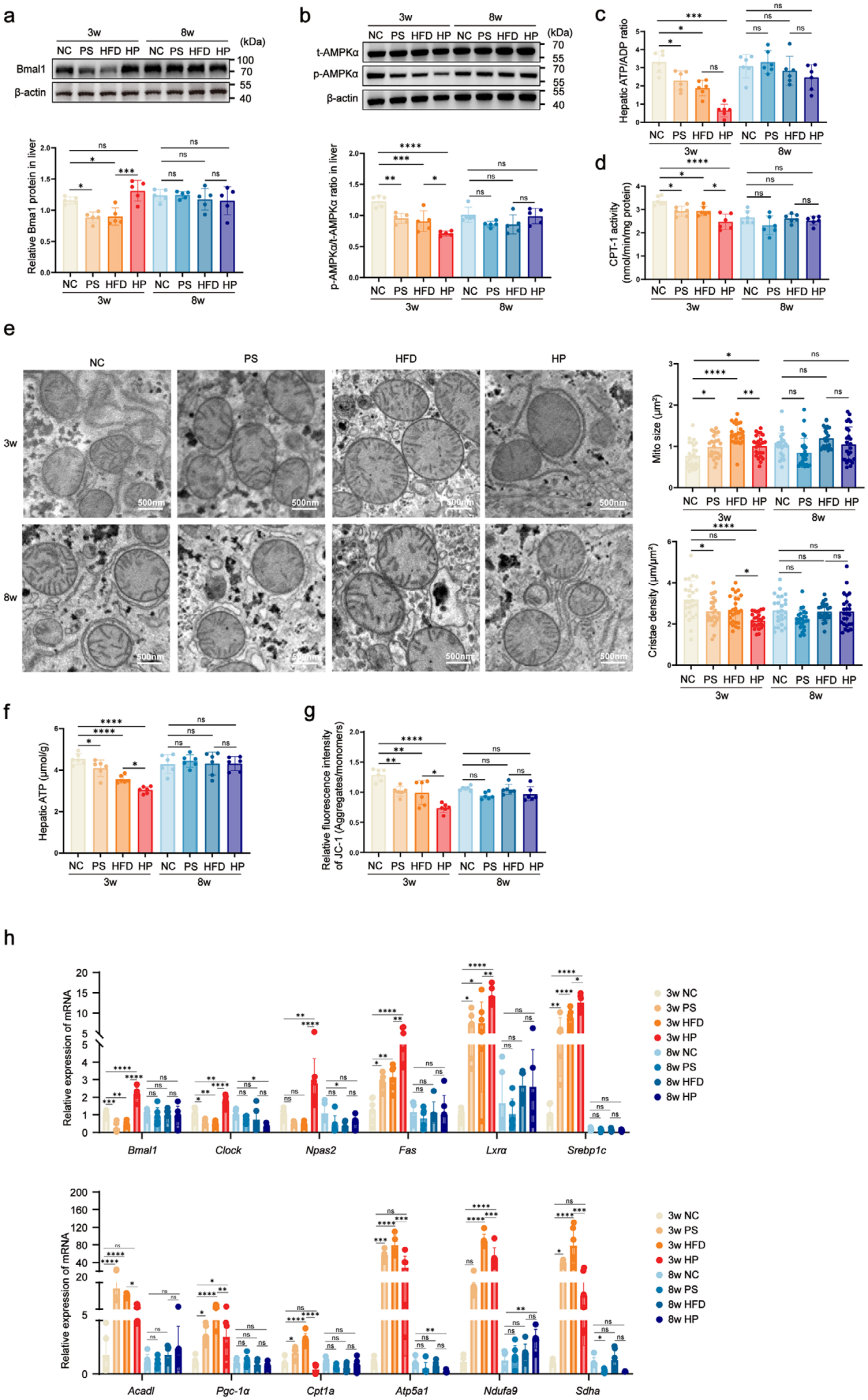
Combined PS‐NPs and HFD exposure disrupt circadian regulation and mitochondrial metabolic function in the juvenile liver. (a) Representative immunoblot images and quantification of hepatic BMAL1 protein abundance. (b) Hepatic AMPK signaling activity evaluated by the levels of t‐AMPKα and p‐AMPKα. (c) Hepatic ATP/ADP ratio. (d) Hepatic carnitine palmitoyltransferase 1 (CPT1) activity in juvenile and adult mice. (e) Representative transmission electron microscopy (TEM) images of mitochondrial ultrastructure and quantitative analysis of mitochondrial area and cristae density. Scale bar, 500 nm. (f) Hepatic ATP content. (g) Mitochondrial membrane potential determined by JC‐1 staining and represented as the fluorescence intensity ratio of aggregates to monomers. (h) Relative mRNA expression levels of representative circadian clock genes (*Bmal1*, *Clock*, and *Npas2*), lipogenic genes (*Fas*, *Lxrα*, and *Srebp1c*), and mitochondrial metabolism‐related genes (*Acadl*, *Pgc‐1α*, *Cpt1a*, *Atp5a1*, *Ndufa9*, and *Sdha*). Data are shown as mean ± SD (n = 5 for a,b; n = 6 for c–g). Two‐way ANOVA (HFD × PS‐NPs) with Tukey correction was used. *p < 0.05, **p < 0.01, ***p < 0.001, ****p < 0.0001; ns, not significant. NC, normal chow; PS, PS‐NPs; HFD, high‐fat diet; HP, HFD + PS‐NPs.

To determine whether the transcriptomic changes were accompanied by functional disturbances, hepatic circadian regulation and mitochondrial energy metabolism were further investigated. Western blot analysis revealed that BMAL1 protein expression was decreased following either PS‐NPs or HFD exposure alone. However, combined exposure resulted in increased BMAL1 abundance at the examined time point (Figure 4a). Given the dynamic nature of circadian oscillations, this increase likely reflects altered circadian phase, compensatory feedback regulation, or temporal misalignment rather than restoration of normal circadian function.

Consistent with impaired circadian‐metabolic coordination, PS‐NPs and HFD exposure progressively disrupted hepatic energy‐sensing pathways. Phosphorylation of AMPK was reduced by either single exposure and further suppressed under combined exposure conditions (Figure 4b). This alteration was accompanied by a decreased hepatic ATP/ADP ratio (Figure 4c), suggesting compromised energy homeostasis and reduced metabolic adaptability. As carnitine palmitoyltransferase 1 (CPT1) serves as the rate‐limiting transporter responsible for mitochondrial uptake of long‐chain fatty acids during β‐oxidation, hepatic CPT1 activity was evaluated as an indicator of mitochondrial fatty acid utilization. In juvenile mice, combined PS‐NPs and HFD exposure significantly decreased CPT1 activity compared with HFD exposure alone, whereas the reduction was modest and statistically insignificant in adult mice (Figure 4d). These results indicate that the juvenile liver exhibits greater impairment in mitochondrial fatty acid transport and oxidative capacity following combined environmental and dietary stress.

Given the close relationship between circadian regulation and mitochondrial function, mitochondrial morphology and bioenergetic performance were subsequently examined. Transmission electron microscopy revealed mitochondrial swelling after either PS‐NPs or HFD exposure alone, whereas combined exposure induced reduced cristae density accompanied by partial alleviation of mitochondrial swelling (Figure 4e), reflecting abnormal mitochondrial structural remodeling. Functional assessments further demonstrated that combined exposure markedly impaired mitochondrial activity, as evidenced by reduced mitochondrial membrane potential and decreased hepatic ATP production compared with either single treatment (Figure 4f,g). Notably, these mitochondrial structural and functional abnormalities were substantially attenuated in adult mice, highlighting the greater sensitivity of the juvenile liver.

At the transcriptional level, combined exposure induced coordinated alterations in genes associated with circadian regulation and hepatic metabolism (Figure 4h). Expression of lipogenic genes, including *Fasn*, *Lxra*, and *Srebp1c*, was progressively elevated following PS‐NPs and HFD co‐exposure. In contrast, genes involved in mitochondrial FAO and oxidative phosphorylation, including *Acadl*, *Pgc‐1α*, *Cpt1a*, *Atp5a1*, *Ndufa9*, and *Sdha*, displayed a distinct biphasic pattern, with increased expression following individual stressors but decreased expression after combined exposure. This transition suggests a shift from an adaptive metabolic response under isolated stress to impaired mitochondrial metabolic regulation under combined environmental and dietary challenges.

Comparative analysis between age groups demonstrated that alterations in circadian regulation, AMPK signaling, and mitochondrial metabolism were substantially more pronounced in juvenile than adult mice. Together, these findings indicate that developmental stage critically determines hepatic susceptibility to PS‐NPs‐associated metabolic disruption, with the immature liver exhibiting enhanced vulnerability to combined nanoplastic and dietary stress.

### 2.4 PS‐NPs Potentiate Lipotoxicity‐Induced Circadian Disruption and Mitochondrial Dysfunction in Hepatocytes In Vitro

**FIGURE 5:**
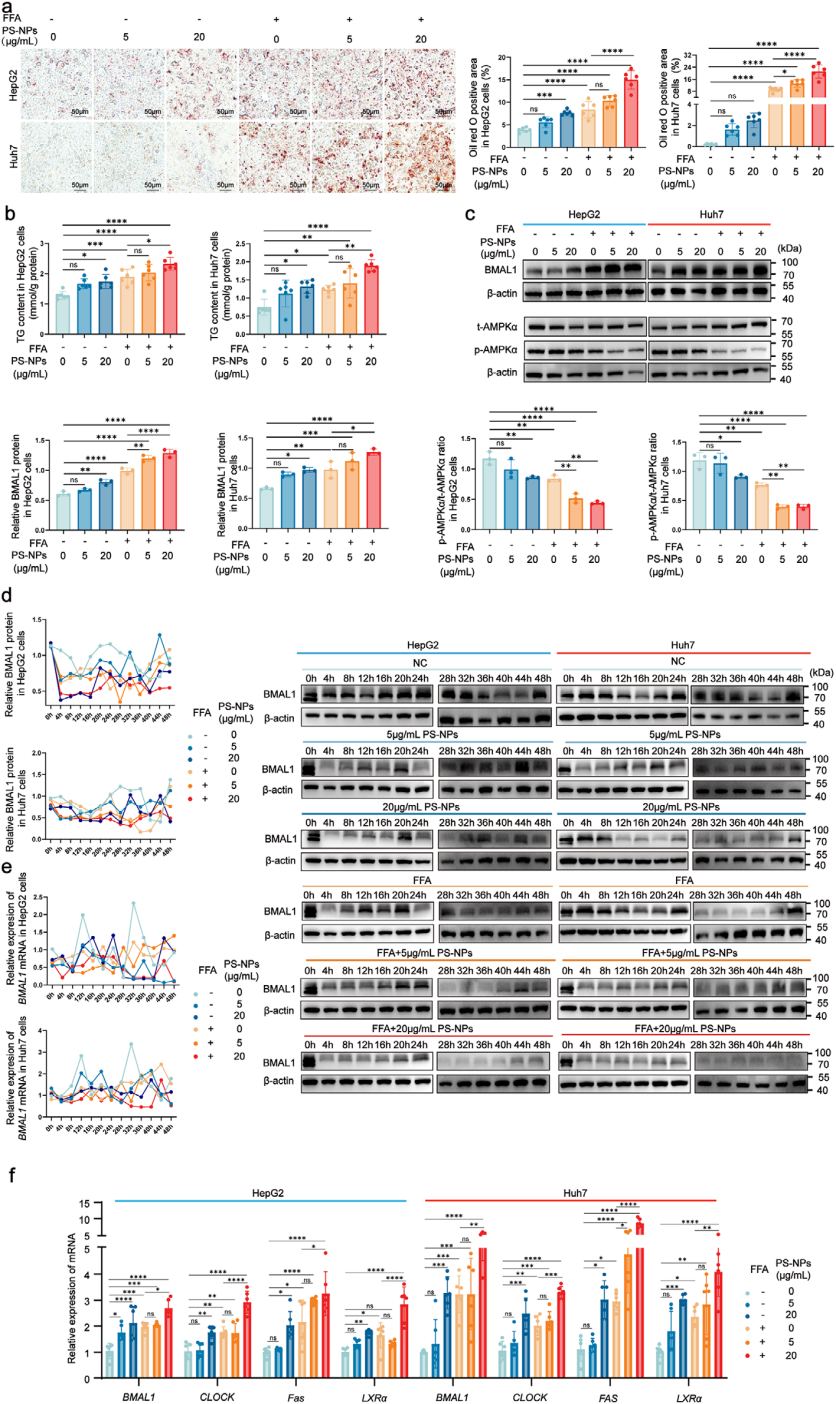
PS‐NPs exacerbate FFA‐induced circadian disruption and lipid accumulation in hepatocyte models. (a) Representative Oil Red O staining images of HepG2 and Huh7 cells treated with FFAs, PS‐NPs, or combined exposure for 48 h. Scale bars, 50 µm. (b) Quantification of Oil Red O‐positive areas and intracellular TG levels. (c) Representative immunoblots and densitometric quantification of BMAL1, t‐AMPKα and p‐AMPKα protein levels after 48 h of treatment. Cells were exposed to an FFA mixture containing 500 µM sodium oleate and 250 µM sodium palmitate, with or without PS‐NPs (5 or 20 µg/mL). (d) Circadian oscillation profiles of BMAL1 protein expression after dexamethasone synchronization (150 nM). Following synchronization, cells were subjected to the indicated treatments and harvested at 4 h intervals over a 48 h period. (e) Time‐dependent changes in BMAL1 mRNA expression after dexamethasone synchronization, with samples collected every 4 h from 0 to 48 h. (f) Relative mRNA expression levels of *BMAL1*, *CLOCK*, *FAS*, and *LXRα* after 48 h of treatment. Data are shown as mean ± SD (n = 3 for c,d; n = 6 for e,f). Panels a, b, f were analyzed by two‐way ANOVA (FFA × PS‐NPs) with Tukey correction. Time‐course data in d, e were analyzed by two‐way ANOVA (treatment × time) with Tukey‐adjusted comparisons. *p < 0.05, **p < 0.01, ***p < 0.001, ****p < 0.0001; ns, not significant.

To determine whether PS‐NPs directly enhance hepatocyte susceptibility to lipotoxic stress, HepG2 and Huh7 cells were co‐exposed to PS‐NPs and free fatty acids (FFAs; 500 µM sodium oleate plus 250 µM sodium palmitate). Compared with FFA treatment alone, co‐exposure significantly increased intracellular lipid accumulation and TG content (Figure 5a,b), indicating aggravated lipotoxic injury.

Consistent with these metabolic changes, co‐exposure induced marked disruption of circadian regulation. Both PS‐NPs and FFAs alone increased BMAL1 protein abundance and reduced AMPK phosphorylation, whereas the most pronounced effects were observed under co‐exposure conditions (Figure 5c). To further assess hepatocyte‐autonomous circadian dynamics, HepG2 and Huh7 cells were synchronized with dexamethasone and BMAL1 expression was monitored over 48 h. In control cells, BMAL1 exhibited robust oscillatory patterns across two circadian cycles. In contrast, PS‐NPs or FFAs alone altered BMAL1 rhythmicity, whereas co‐exposure caused more pronounced circadian disruption, characterized by blunted oscillatory amplitude and sustained suppression of BMAL1 protein, particularly under FFA plus 20 µg/mL PS‐NPs conditions (Figure 5d). Consistently, *BMAL1* mRNA time‐course analysis revealed altered temporal expression dynamics following PS‐NPs and/or FFA exposure, further supporting disruption of hepatocyte circadian regulation under combined stress (Figure 5e). In addition, co‐exposure altered the expression of key circadian and metabolic regulators, including *BMAL1*, *CLOCK*, *FAS*, and *LXRα* (Figure 5f), indicating coordinated remodeling of clock‐controlled metabolic programs.

**FIGURE 6:**
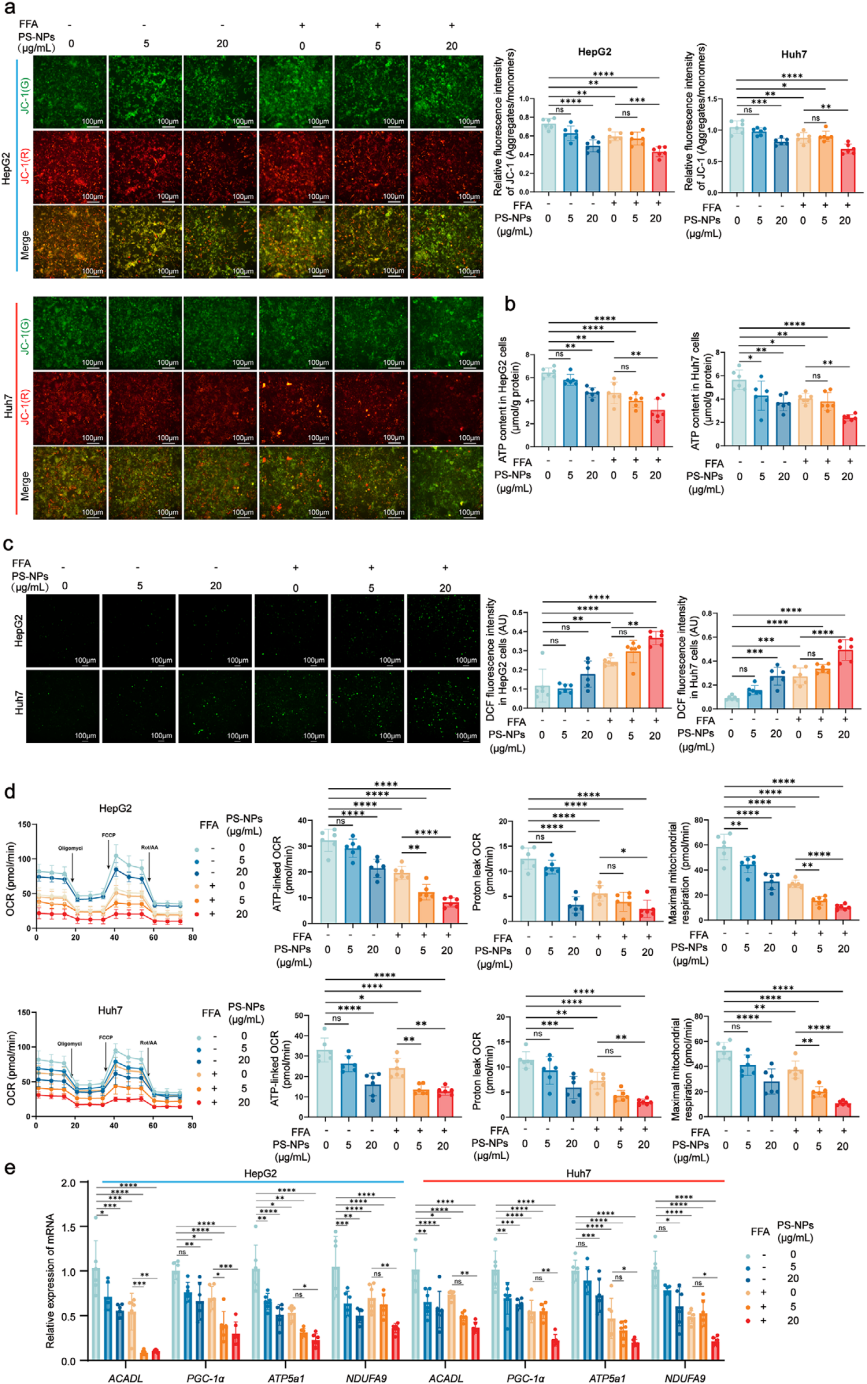
PS‐NPs exacerbate FFA‐induced mitochondrial dysfunction and bioenergetic impairment in hepatocytes in vitro. (a) Representative JC‐1 fluorescence images showing mitochondrial membrane potential alterations. Scale bars, 100 µm. (b) Intracellular ATP levels in hepatocytes after 48 h exposure to FFAs, PS‐NPs (5 or 20 µg/mL), or combined treatment. (c) Intracellular ROS generation assessed by H_2_DCFDA fluorescence staining. Scale bars, 100 µm. (d) Seahorse analysis of basal, ATP‐linked, proton leak, and maximal respiration. (e) Relative mRNA expression levels of *Acadl*, *Pgc‐1α*, *Atp5a1*, and *Ndufa9*. Data are shown as mean ± SD (n = 6). Two‐way ANOVA (HFD × PS‐NPs) with Tukey correction was used. *p < 0.05, **p < 0.01, ***p < 0.001, ****p < 0.0001; ns, not significant.

Given the tight coupling between circadian regulation and mitochondrial metabolism, mitochondrial function was next evaluated. Co‐exposure significantly reduced mitochondrial membrane potential and ATP production, while increasing intracellular reactive oxygen species (ROS) levels (Figure 6a–c). Seahorse mitochondrial stress analysis further demonstrated a broad suppression of mitochondrial respiratory capacity under PS‐NPs and FFA co‐exposure (Figure 6d). Specifically, ATP‐linked oxygen consumption rate (OCR), reflecting oligomycin‐sensitive respiration coupled to ATP synthesis, was markedly decreased, consistent with reduced intracellular ATP levels. Maximal respiration, assessed following carbonyl cyanide‐p‐trifluoromethoxyphenylhydrazone (FCCP) stimulation, was also reduced, indicating impaired electron transport chain capacity and diminished mitochondrial respiratory reserve. In addition, absolute proton leak OCR was decreased rather than increased; as proton leak was reported as an absolute OCR value, this reduction should be interpreted cautiously and likely reflects an overall decline in mitochondrial respiratory activity rather than uncoupling. Consistently, genes involved in mitochondrial fatty acid β‐oxidation and oxidative metabolism (*Acadl*, *Pgc‐1α*) as well as oxidative phosphorylation (*Atp5a1*, *Ndufa9*) were downregulated under co‐exposure conditions (Figure 6e), supporting progressive mitochondrial bioenergetic failure.

Altogether, these findings demonstrate that PS‐NPs directly exacerbate FFA‐induced lipotoxicity by disrupting circadian regulation, suppressing AMPK‐dependent energy sensing, and impairing mitochondrial bioenergetic function, thereby linking circadian dysregulation to metabolic deterioration in hepatocytes.

### 2.5 AMPK Activation Rescues PS‐NPs‐Induced Metabolic Dysfunction in Hepatocytes In Vitro

**FIGURE 7:**
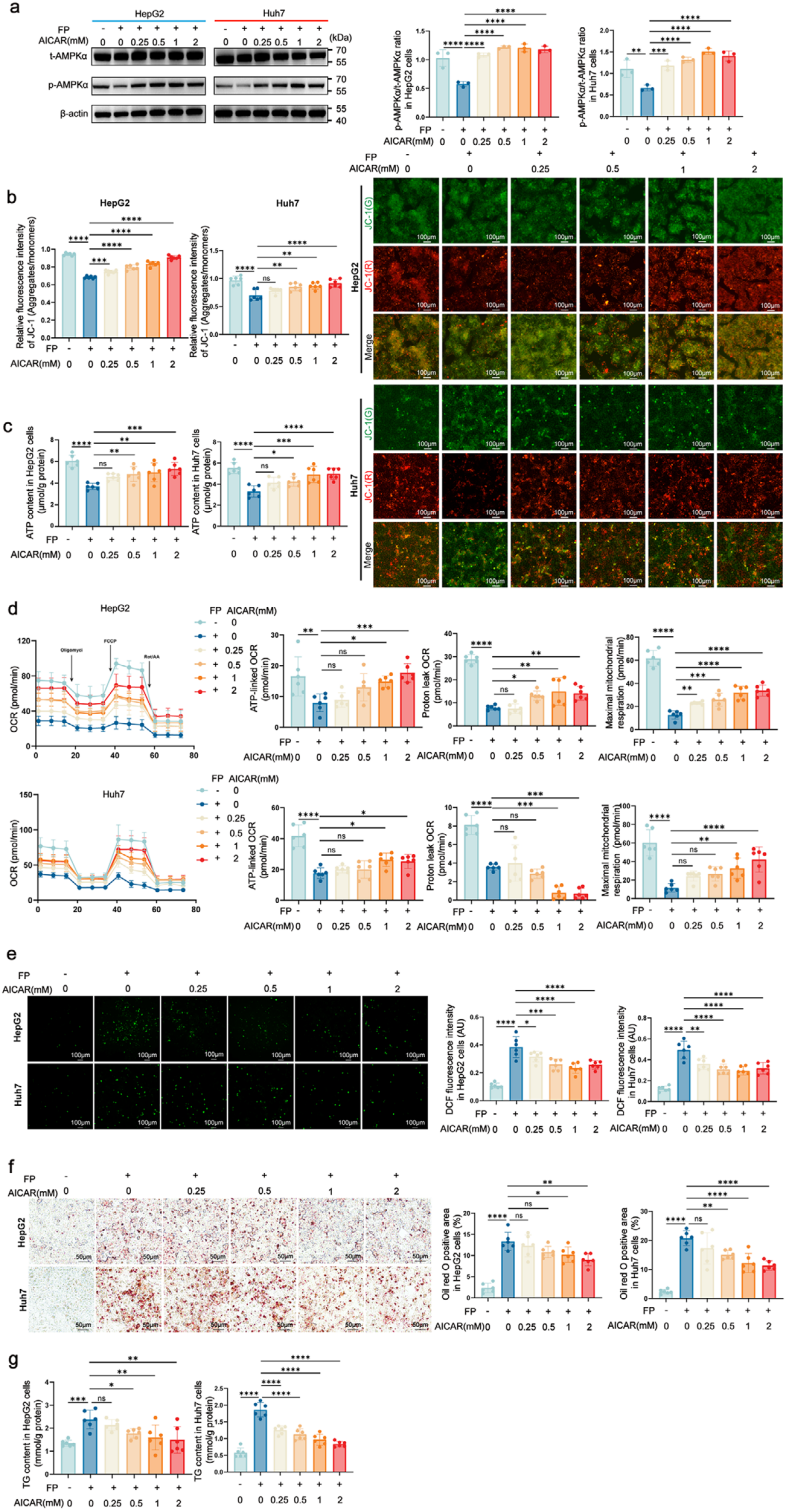
Pharmacological activation of AMPK by AICAR mitigates PS‐NPs‐ and FFA‐induced metabolic disturbances in hepatocytes in vitro. (a) Dose‐dependent activation of AMPK following 48 h exposure to AICAR (0.25–2 mM), assessed by phosphorylated AMPKα (p‐AMPKα) protein abundance. (b) JC‐1 images showing mitochondrial membrane potential after AICAR treatment. Scale bar, 100 µm. (c) Quantification of intracellular ATP levels. (d) Seahorse analysis of ATP‐linked respiration, proton leak, and maximal respiration. (e) Intracellular ROS levels measured by H_2_DCFDA fluorescence staining. Scale bars, 100 µm. (f) Representative Oil Red O staining images and quantification of intracellular lipid accumulation. Scale bars, 50 µm. (g) Measurement of intracellular TG content. Data are shown as mean ± SD (n = 3 for a; n = 6 for b–g). One‐way ANOVA with Tukey correction was used. *p < 0.05, **p < 0.01, ***p < 0.001, and ****p < 0.0001; ns, not significant.

Given the pronounced suppression of AMPK signaling under co‐exposure conditions, we next examined whether pharmacological activation of AMPK could restore hepatocellular metabolic homeostasis in vitro. Treatment with AICAR dose‐dependently increased AMPK phosphorylation in both HepG2 and Huh7 cells (Figure 7a).

Restoration of AMPK activity markedly improved mitochondrial function. AICAR significantly increased mitochondrial membrane potential and ATP production compared with the co‐exposure group (Figure 7b,c). Consistently, Seahorse flux analysis demonstrated partial recovery of mitochondrial respiratory capacity, as evidenced by increased ATP‐linked OCR and maximal respiration (Figure 7d). Proton leak OCR was also increased in HepG2 following AICAR treatment, which likely reflects a generalized restoration of mitochondrial respiratory activity rather than enhanced uncoupling.

Improved mitochondrial bioenergetics was accompanied by attenuation of oxidative stress. AICAR treatment markedly reduced PS ‐NPs‐and FFA‐induced ROS accumulation (Figure 7e), indicating restoration of intracellular redox balance.

These mitochondrial and redox improvements translated into clear metabolic benefits. AICAR significantly reduced intracellular lipid droplet accumulation and TG content compared with untreated co‐exposed cells (Figure 7f,g), indicating alleviation of lipotoxic injury.

As a whole, AMPK activation effectively mitigated PS‐NPs‐enhanced lipotoxicity by restoring mitochondrial bioenergetic function, improving redox homeostasis, and reducing lipid accumulation, underscoring a central role for impaired AMPK signaling in mediating PS‐NPs‐driven metabolic dysfunction under lipotoxic stress conditions.

### 2.6 Circadian Modulation Partially Alleviates PS‐NPs‐Enhanced Metabolic Dysfunction in Hepatocytes In Vitro

**FIGURE 8:**
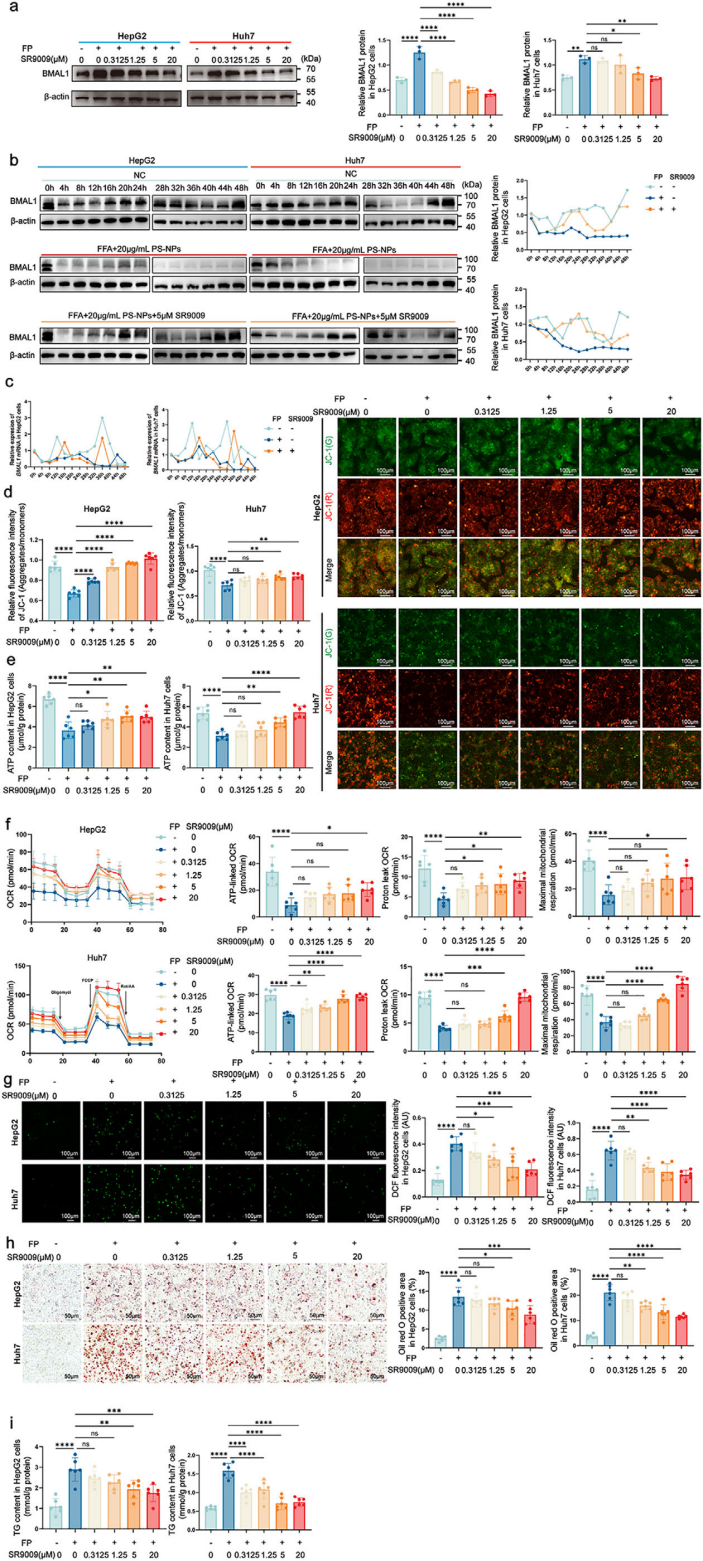
SR9009‐mediated circadian regulation ameliorates mitochondrial dysfunction and metabolic imbalance in hepatocytes in vitro. (a) Dose‐dependent effects of SR9009 (0.3125‐20 µM) on BMAL1 protein abundance after 48 h treatment. (b) Temporal oscillation patterns of BMAL1 expression following dexamethasone synchronization (150 nM). Cells were collected at 4 h intervals from 0 to 48 h (0, 4, 8, 12, 16, 20, 24, 28, 32, 36, 40, 44, and 48 h). (c) Quantitative analysis of BMAL1 rhythmicity based on time‐series mRNA expression profiles. (d) JC‐1 assessment of mitochondrial membrane potential using the aggregate/monomer fluorescence ratio. Scale bar, 100 µm. (e) Quantification of intracellular ATP levels. (f) Mitochondrial respiratory parameters determined using Seahorse XF extracellular flux analysis. (g) Intracellular ROS levels assessed by H_2_DCFDA fluorescence staining. Scale bars, 100 µm. (h) Representative Oil Red O staining images of HepG2 and Huh7 cells. Scale bars, 50 µm. (i) Quantification of intracellular TG content. Data are shown as mean ± SD (n = 3 for a,b; n = 6 for c–i). Panels a and d–i were analyzed by one‐way ANOVA with Tukey correction. Time‐course BMAL1 data in b,c were analyzed by two‐way ANOVA (treatment × time) with Tukey‐adjusted comparisons. *p < 0.05, **p < 0.01, ***p < 0.001, and ****p < 0.0001; ns, not significant.

To determine whether circadian disruption directly contributes to PS‐NPs‐enhanced lipotoxicity, hepatocytes co‐exposed to PS‐NPs and FFAs were treated with the REV‐ERB agonist SR9009. SR9009 induced a dose‐dependent decrease in BMAL1 protein abundance, confirming effective circadian modulation (Figure 8a). Following dexamethasone synchronization, co‐exposure markedly attenuated the amplitude and altered the phase of BMAL1 oscillation. SR9009 partially restored both oscillatory amplitude and phase dynamics, indicating that PS‐NPs‐induced circadian disruption is at least partially reversible (Figure 8b,c).

Restoration of circadian rhythmicity was accompanied by improved mitochondrial function. SR9009 treatment increased mitochondrial membrane potential and ATP production (Figure 8d,e). Consistently, Seahorse extracellular flux analysis revealed enhanced ATP‐linked respiration, maximal respiration, spare respiratory capacity, and coupling efficiency, together with normalization of proton leak (Figure 8f). In parallel, intracellular ROS levels were markedly reduced (Figure 8g), indicating restoration of redox homeostasis.

These mitochondrial and redox improvements translated into metabolic benefits. SR9009 significantly reduced intracellular lipid accumulation and TG content compared with untreated co‐exposed cells (Figure 8h,i), indicating partial alleviation of lipotoxic injury.

Together, circadian modulation partially rescues PS‐NPs‐enhanced metabolic dysfunction by restoring BMAL1 rhythmicity, improving mitochondrial bioenergetics, and reducing lipid accumulation. The coordinated recovery of circadian, mitochondrial, and metabolic parameters supports a mechanistic role for circadian disruption in mediating PS‐NPs‐induced lipotoxicity.

### 2.7 AMPK Activation and Circadian Modulation Confer Complementary Protection In Vivo

**FIGURE 9:**
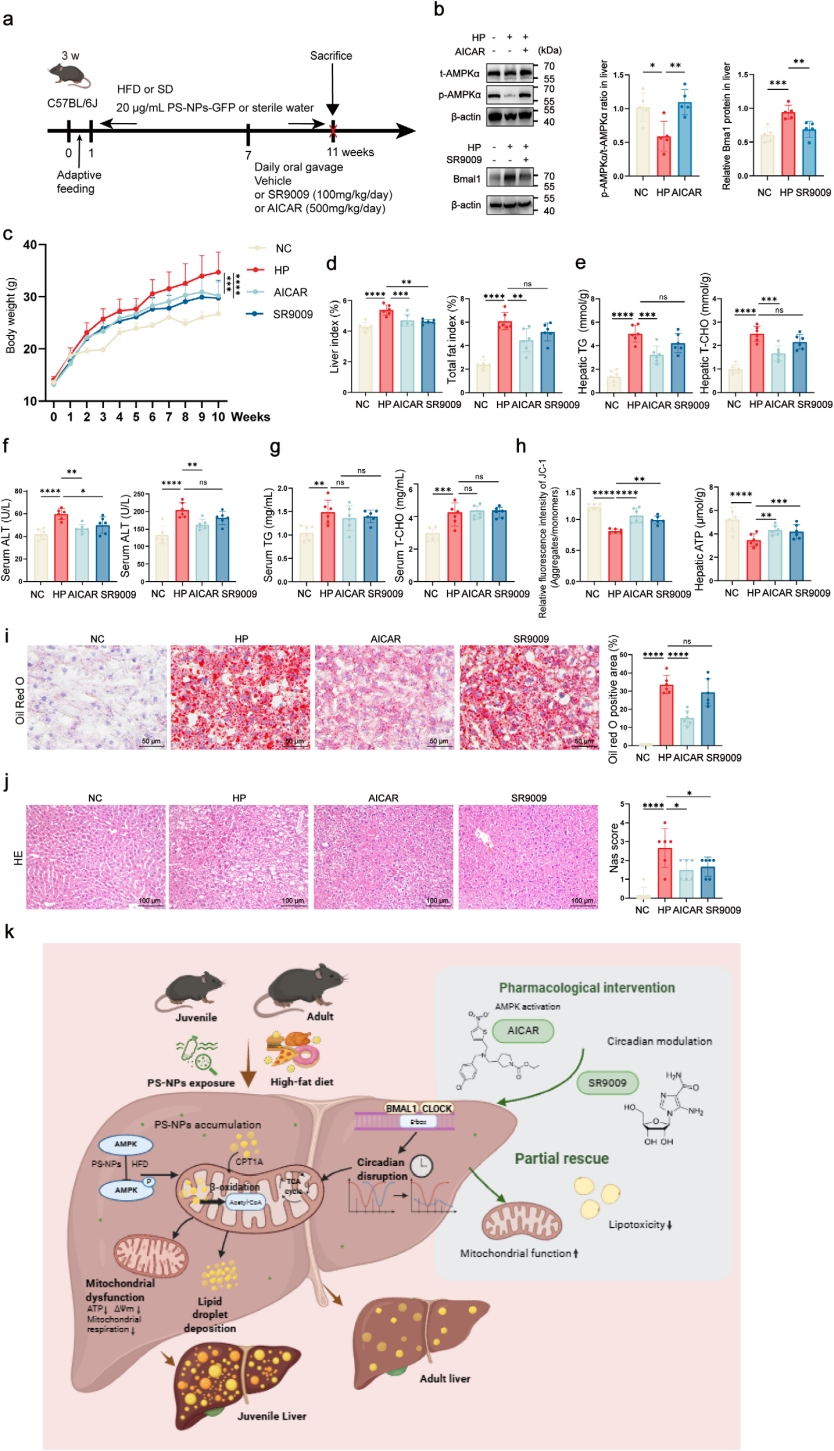
AMPK activation and circadian modulation provide complementary metabolic benefits against PS‐NPs‐ and HFD‐induced hepatic dysfunction in vivo. (a) Experimental design of pharmacological intervention. Mice receiving combined HFD and PS‐NPs exposure were treated with AICAR (500 mg/kg/day) or SR9009 (100 mg/kg/day) by oral gavage during the final 4 weeks of the 10week exposure protocol. (b) Validation of target engagement: hepatic phosphorylated AMPKα (p‐AMPKα) and total AMPKα levels following AICAR administration, and hepatic BMAL1 protein abundance following SR9009 treatment. (c) Changes in body weight during the intervention period. (d) Liver and adipose tissue indices. (e) Hepatic TG and T‐CHO contents. (f) Serum ALT and AST activities. (g) Serum TG and T‐CHO concentrations. (h) Hepatic mitochondrial membrane potential assessed by JC‐1 staining and hepatic ATP content. (i) Representative Oil Red O‐stained liver sections and quantification of lipid‐positive areas. Scale bars, 50 µm. (j) Representative H&E‐stained liver sections and corresponding NAS. Scale bars, 100 µm. (k) Schematic illustration of the proposed mechanism. PS‐NPs aggravate HFD‐associated hepatic metabolic dysfunction through disruption of circadian regulation, mitochondrial homeostasis, and AMPK signaling. AMPK activation predominantly improves hepatic lipid metabolism, whereas circadian modulation primarily enhances mitochondrial functional recovery. Created with BioRender. Xu, P. (2026). https://BioRender.com/o0rrsac (Agreement No. IS2A7Q9BHX). Data are shown as mean ± SD (n = 5 for b; n = 6 for c–j). One‐way ANOVA with Tukey correction was used. *p < 0.05, **p < 0.01, ***p < 0.001, and ****p < 0.0001; ns, not significant. NC, normal chow; PS, PS‐NPs; HFD, high‐fat diet; HP, HP, HFD + PS‐NPs.

To delineate the distinct contributions of AMPK signaling and circadian regulation in vivo, juvenile mice co‐exposed to PS‐NPs and HFD were treated with AICAR (500 mg/kg/day) or SR9009 (100 mg/kg/day), both administered via oral gavage, during the final 4 weeks of exposure (Figure 9a). Target engagement was confirmed by increased hepatic AMPK phosphorylation following AICAR administration and reduced BMAL1 abundance after SR9009 treatment (Figure 9b).

The metabolic effects of AICAR and SR9009 intervention displayed distinct phenotypic profiles, suggesting differential modes of metabolic regulation. Both treatments significantly reduced HFD plus PS‐NPs‐induced body weight gain (Figure 9c). AICAR treatment produced a pronounced improvement in metabolic abnormalities, including reductions in liver index, adiposity index, and hepatic lipid accumulation. Although SR9009 also decreased liver index, its effects on adiposity and hepatic steatosis were comparatively limited (Figure 9d,e). Consistent with these findings, AICAR significantly lowered serum ALT and AST levels, whereas SR9009 reduced ALT levels without significantly affecting AST, indicating differential capacities of AMPK activation and circadian modulation in mitigating hepatocellular injury (Figure 9f). In contrast, neither intervention significantly altered circulating TG or T‐CHO concentrations, suggesting that their beneficial effects were primarily manifested within hepatic metabolic regulation rather than systemic lipid homeostasis (Figure 9g).

Despite their distinct effects on lipid accumulation, both AICAR and SR9009 improved mitochondrial functional parameters, as evidenced by enhanced mitochondrial membrane potential and increased hepatic ATP production. Notably, SR9009 exerted a robust protective effect on mitochondrial bioenergetics despite its relatively modest impact on hepatic steatosis, highlighting a partial dissociation between mitochondrial functional recovery and lipid deposition (Figure 9h). Histological examination further confirmed that AICAR markedly reduced hepatic lipid droplet accumulation, whereas SR9009 produced a milder improvement. Nevertheless, both interventions partially alleviated hepatic pathological changes, as indicated by decreased NAFLD activity scores (NAS) (Figure 9i,j).

The observed dissociation between the lipid‐lowering efficacy of AICAR and the prominent mitochondrial protection conferred by SR9009 supports a distributed regulatory network model in which AMPK‐mediated energy sensing, REV‐ERB/BMAL1‐dependent circadian pathways, and mitochondrial metabolism function as interconnected yet partially independent nodes. Within this framework, AMPK activation primarily mitigates hepatic lipid deposition, whereas circadian modulation predominantly enhances mitochondrial integrity and cellular resilience.

In summary, these findings demonstrate that AMPK activation and circadian modulation confer distinct but complementary protection against PS‐NPs‐ and HFD‐induced hepatic metabolic dysfunction. They further indicate that PS‐NPs act as age‐dependent metabolic disruptors, amplifying HFD‐induced steatosis in juvenile mice through coordinated perturbation of the circadian‐mitochondrial‐AMPK axis, with partial reversal achievable via targeting discrete regulatory nodes (Figure 9k).

## 3 Discussion

Nanoplastics have emerged as persistent environmental contaminants with increasing concerns regarding their potential effects on human metabolic health. In this study, we demonstrate that chronic oral exposure to PS‐NPs markedly exacerbates HFD‐induced hepatic steatosis in juvenile mice, whereas adult mice exhibit substantially lower susceptibility despite comparable caloric intake, body weight‐normalized PS‐NP exposure, and hepatic accumulation. These findings indicate that developmental stage, rather than exposure burden alone, represents a critical determinant of susceptibility to nanoplastic‐associated metabolic disruption. Consistent with the DOHaD framework [13, 26], environmental insults encountered during sensitive developmental windows may induce more profound and persistent metabolic consequences than comparable exposures occurring after maturation. The heightened vulnerability of juvenile mice may arise from the incomplete maturation of hepatic circadian regulation, mitochondrial metabolic networks, and nutrient‐sensing pathways, which collectively limits adaptive capacity in response to concurrent dietary and environmental stressors.

Previous studies have demonstrated that PS‐NPs can promote hepatic lipid accumulation through diverse mechanisms, including impaired autophagy, altered nuclear receptor signaling, and disrupted lipid metabolism [15, 16]. However, whether developmental stage modifies susceptibility to PS‐NPs‐associated metabolic injury and how environmental exposure integrates into hepatic regulatory networks remain insufficiently understood. Our findings extend current knowledge by demonstrating that early‐life exposure represents a critical window during which PS‐NPs amplify diet‐induced metabolic dysfunction. Importantly, the aggravated phenotype in juvenile mice was not attributable to increased PS‐NPs exposure, excessive caloric intake, or enhanced hepatic accumulation, suggesting that intrinsic developmental differences in metabolic regulation contribute substantially to disease susceptibility.

Mechanistically, PS‐NPs‐induced metabolic dysfunction was associated with coordinated disturbances in circadian regulation, mitochondrial homeostasis, and AMPK‐dependent energy sensing. The hepatic circadian clock orchestrates daily oscillations in lipid synthesis, fatty acid oxidation, mitochondrial activity, and nutrient utilization, thereby maintaining metabolic flexibility in response to feeding‐fasting cycles [17, 18, 19, 20, 21]. In the present study, altered hepatic BMAL1 expression following PS‐NPs and HFD co‐exposure suggested disruption of circadian regulation; however, interpretation of this single‐time‐point measurement requires caution because circadian function is determined by dynamic parameters, including phase, amplitude, and periodicity. In vitro synchronization experiments further demonstrated altered BMAL1 oscillatory patterns, although immortalized hepatocyte models cannot fully reproduce systemic circadian entrainment signals derived from hormonal, neural, and feeding cues. Therefore, comprehensive temporal profiling in vivo will be required to further define the impact of PS‐NPs on hepatic circadian organization [27, 28].

Notably, the non‐linear transcriptional responses of key circadian regulators, including *Bmal1* and *Npas2*, suggest that altered expression patterns may reflect phase‐dependent regulatory remodeling rather than restoration of circadian homeostasis. In addition, the persistent faster‐migrating BMAL1 protein band observed in this study raises the possibility of altered post‐translational modification or protein processing. Although such modifications have been implicated in circadian regulation, the molecular mechanisms underlying this observation remain to be determined. Future studies integrating temporal proteomics and post‐translational modification analyses may provide additional insight into how nanoplastic exposure alters circadian machinery [29, 30].

Circadian disruption was accompanied by profound mitochondrial dysfunction [31, 32]. Although transcriptomic analyses revealed enrichment of oxidative phosphorylation and fatty acid metabolism pathways, functional assays demonstrated impaired mitochondrial membrane potential, reduced ATP production, decreased respiratory capacity, impaired fatty acid utilization, and increased ROS accumulation. Seahorse analysis further confirmed reductions in basal respiration, ATP‐linked respiration, maximal respiration, and spare respiratory capacity, indicating that transcriptional activation of mitochondrial pathways may represent an adaptive response to energetic stress rather than preserved mitochondrial function. These findings highlight the importance of integrating transcriptomic and functional analyses when evaluating environmental metabolic toxicity.

Consistently, reduced CPT1 activity in hepatic mitochondrial fractions provided additional evidence of impaired mitochondrial fatty acid transport capacity under PS‐NPs‐induced metabolic stress. However, CPT1 activity reflects mitochondrial long‐chain fatty acid import capacity but does not directly represent the dynamic process of mitochondrial FAO [33, 34]. Because the commercial assay used in this study was not validated for isoform‐specific detection, the measured activity was conservatively described as CPT1 activity rather than CPT1A‐specific activity. Future studies incorporating mitochondrial FAO flux assays, isotope tracing, acyl‐carnitine profiling, and direct CPT1A enzymatic measurements will be necessary to further clarify how impaired lipid oxidation contributes to PS‐NPs‐associated hepatic steatosis. Nevertheless, the combined evidence from mitochondrial structural, biochemical, and transcriptional analyses supports mitochondrial metabolic impairment as a major contributor to nanoplastic‐induced hepatic dysfunction.

AMPK emerged as an important regulatory component linking energy sensing with mitochondrial metabolic adaptation. AMPK functions as a central cellular energy sensor that coordinates nutrient availability, mitochondrial activity, and lipid metabolism [22, 23]. In this study, PS‐NPs and HFD co‐exposure suppressed AMPK phosphorylation, accompanied by impaired mitochondrial bioenergetics and enhanced hepatic lipid accumulation. Pharmacological activation of AMPK using AICAR restored mitochondrial membrane potential, ATP production, redox balance, and hepatic lipid homeostasis, consistent with previous reports implicating suppression of the SIRT1‐AMPK pathway in PS‐NPs‐induced metabolic stress. These findings suggest that AMPK signaling represents an important adaptive pathway counteracting nanoplastic‐induced metabolic imbalance [35].

Pharmacological rescue experiments further revealed functional differences among metabolic regulatory modules. AICAR exerted a stronger effect on reducing hepatic lipid accumulation and improving steatosis‐associated phenotypes, whereas SR9009 primarily enhanced mitochondrial function and partially restored BMAL1 rhythmicity without completely reversing lipid deposition. Similarly, SR9011, another REV‐ERB agonist, improved mitochondrial parameters, supporting a contribution of circadian regulatory pathways to PS‐NPs‐associated metabolic dysfunction (Figure S4a–d). Together, these findings support a distributed regulatory network in which AMPK‐mediated energy sensing, REV‐ERB/BMAL1‐driven circadian regulation, and mitochondrial metabolism are interconnected but partially independent components [36]. However, pharmacological approaches should be interpreted cautiously, as off‐target or pathway‐independent effects of AICAR and REV‐ERB agonists cannot be completely excluded [37]. Genetic validation using tissue‐specific loss‐of‐function or gain‐of‐function models will be essential to establish definitive causal relationships.

The exposure paradigm used in this study was designed to simulate sustained low‐level oral PS‐NPs exposure while minimizing overt systemic toxicity. Comparable biological responses between GFP‐labeled and unlabeled PS‐NPs suggest that fluorescent labeling did not substantially influence the observed metabolic effects. Nevertheless, several limitations should be considered. Because mice were group‐housed and exposed through drinking water, individual PS‐NPs intake could not be directly quantified. Exposure levels were therefore estimated based on cage‐level water consumption, animal number, and body weight normalization. Although this approach maintains physiological relevance for chronic environmental exposure scenarios, it cannot fully exclude individual differences in drinking behavior, gastrointestinal absorption, biodistribution, and tissue accumulation. Single housing or oral gavage administration could improve dose precision but may introduce stress‐related metabolic alterations or reduce the ecological relevance of continuous environmental exposure. Future studies incorporating automated intake monitoring, metabolic cage systems, or individual exposure tracking will improve exposure quantification and enable more accurate dose‐response analysis.

Several additional limitations should also be acknowledged. First, only one PS‐NPs formulation, particle size, and exposure concentration were investigated. Given the substantial heterogeneity of environmental nanoplastics, including differences in polymer composition, size distribution, surface characteristics, aging status, and co‐contaminant interactions, whether these findings extend to other nanoplastic types or mixed exposure scenarios remains uncertain. Second, although the selected exposure concentration is within the range commonly used in experimental studies, its quantitative relevance to real‐world human exposure requires further evaluation using environmentally representative concentrations and exposure mixtures. Third, circadian alterations were primarily characterized using selected time points and in vitro models, limiting comprehensive evaluation of long‐term rhythmic changes in vivo. Finally, this study was performed exclusively in male mice; therefore, potential sex‐dependent differences in susceptibility and metabolic responses remain to be investigated.

Despite these limitations, the present study provides evidence that PS‐NPs function as developmental metabolic stressors that increase susceptibility to HFD‐induced hepatic steatosis through coordinated disruption of circadian regulation, mitochondrial metabolism, and AMPK‐dependent energy sensing. Identification of an early‐life vulnerability window and characterization of the interconnected circadian‐mitochondrial metabolic network provide new mechanistic insight into nanoplastic‐induced metabolic disease. These findings further support environmental chronotoxicity as a conceptual framework for understanding how persistent environmental exposures perturb temporal metabolic organization and contribute to the progression of metabolic disorders.

## 4 Methods

### 4.1 Animals

Male C57BL/6J mice were purchased from Shanghai SLAC Laboratory Animal Co., Ltd. Two age cohorts were established using animals obtained at 3 weeks of age and 8 weeks of age, representing the juvenile and adult groups, respectively. The animal room was maintained at 25°C ± 1°C and 50%–60% relative humidity on a 12‐h light/12‐h dark schedule. The light phase began at 06:00, which was defined as Zeitgeber time 0 (ZT0).

The study protocol was reviewed and approved by the Experimental Animal Ethics and Welfare Committee of Zhejiang Chinese Medical University (ethics application No. 2025066; approval no. ZCMUJMJY‐202503‐05). Animal procedures complied with applicable national regulations and guidelines for laboratory animal care and use. In vivo studies were conducted at the Jinhua Academy of Zhejiang Chinese Medical University under institutional animal‐use license SYXK (Zhe) 2024‐0027.

### 4.2 Food and Water Intake Measurement

Because the animals were group‐housed, consumption was recorded for each cage. At the beginning of every 3‐day interval, each cage received 100 g of chow and 200 mL of water. The remaining amounts were measured before both supplies were restored to their starting levels. Consumption was summed by week, divided by the number of mice in the cage, and normalized to body mass. Weekly food and water intake are reported as g/g body weight/week and mL/g body weight/week, respectively. Energy intake was derived from food consumption and the manufacturer‐provided caloric density and expressed as kcal/g body weight.

### 4.3 Cell Culture

HepG2 and Huh7 human hepatocyte cell lines were obtained from the Cell Bank of the Chinese Academy of Sciences and authenticated by short tandem repeat profiling. Cells were propagated in high‐glucose Dulbecco's modified Eagle's medium (DMEM; CellMax, CGM101.05) containing 10% fetal bovine serum (Gibco, B21069SRP) and 1% penicillin‐streptomycin (Biosharp, BL505A). Cultures were kept at 37°C in a humidified incubator containing 5% CO_2_. Mycoplasma contamination was routinely monitored throughout the study to ensure the reliability of experimental results.

### 4.4 PS‐NPs Preparation and Characterization

Fluorescent polystyrene nanoplastics (PS‐NPs‐GFP; nominal diameter 100 nm, FF3301G) were purchased from Rigor Biotechnology. Stock suspensions were vortexed and sonicated (40 kHz, 10 min) prior to use to minimize aggregation and ensure homogeneous dispersion. For in vivo exposure, PS‐NPs were prepared at 20 µg/mL in drinking water, and the exposure solution was replaced every 3 days [15, 16, 38]. To reduce particle sedimentation during administration, drinking water bottles were gently mixed before replacement and sonicated twice daily for 30 min.

Particle morphology and primary size were confirmed by SEM, consistent with the manufacturer's specifications. Particle size distribution and surface charge were characterized with a Zetasizer Nano ZS90 (Malvern Instruments, UK), and the resulting data were processed using Zetasizer Software v7.3.0. DLS showed a hydrodynamic diameter of 115.4 ± 1.5 nm and a PDI of 0.041 ± 0.040. Zeta potential measurements yielded a mean value of −30.9 ± 0.7 mV, consistent with a negatively charged surface and stable aqueous suspension.

To further assess dispersion stability during exposure, drinking water samples were collected daily prior to replacement and examined by SEM. No detectable aggregation or sedimentation was observed over the 3‐day replacement cycle, confirming sustained particle stability under experimental conditions.

### 4.5 High‐Fat Diet and PS‐NPs Exposure

Animals were randomly divided into four treatment groups: control, PS‐NPs, HFD, and combined PS‐NPs/HFD. HFD‐fed mice received a 60% kcal fat diet (Research Diets, D12492), whereas controls received standard chow. PS‐NPs (100 nm) were provided in drinking water at 20 µg/mL for 10 weeks, a concentration chosen based on previous chronic exposure studies and preliminary validation to avoid overt toxicity (Figure S5a).

In vitro, PS‐NPs were applied at 20 µg/mL, independently of in vivo fluorescence data, based on dose‐ranging and cell viability assays. This concentration represents the highest non‐cytotoxic dose, allowing assessment of PS‐NPs‐mediated metabolic and circadian effects while minimizing nonspecific toxicity.

Notably, hepatic GFP fluorescence was used only to confirm biodistribution and relative hepatic accumulation, not to estimate absolute tissue concentrations or guide in vitro dosing.

### 4.6 In Vivo Pharmacological Rescue With AICAR and SR9009

After 6 weeks of HFD and/or PS‐NPs exposure, mice received AICAR (500 mg/kg/day) or SR9009 (100 mg/kg/day) by oral gavage for 4 weeks; controls received vehicle. Dosing was performed at a fixed Zeitgeber time.

### 4.7 In Vitro Pharmacological Rescue With AICAR and SR9009

Hepatocytes were exposed to FFAs and/or PS‐NPs (20 µg/mL) for 48 h to establish the cellular injury model. AICAR (0.25–2 mM, Selleck, S1802) or SR9009 (0.3125–20 µM, MedChemExpress, HY‐16989) was applied throughout the exposure period. Culture medium was refreshed every 24 h to maintain compound stability and activity. Vehicle controls received equivalent volumes of PBS or DMSO (final DMSO ≤ 0.1%). Cells were harvested after 48 h for downstream analyses.

### 4.8 Circadian Control and Zeitgeber Time

Circadian experiments followed the light‐dark cycle, with lights on at 06:00 defined as ZT0. Mice were acclimated for 7 days under a 12‐h light/12‐h dark cycle before experiments. Unless otherwise indicated, metabolic interventions, tolerance tests, euthanasia, and terminal tissue collection were performed within the same circadian window (ZT4‐ZT6) to minimize potential effects of temporal variation. Animals had ad libitum access to food and water except during fasting periods specifically required for metabolic assays. At designated Zeitgeber times, mice were euthanized and liver samples were snap‐frozen in liquid nitrogen and stored at −80°C.

### 4.9 Cellular Circadian Rhythm Assay

HepG2 and Huh7 cells at 70%–80% confluence were synchronized with 150 nM dexamethasone for 2 h [39]. The cells then received fresh complete medium containing the indicated combinations of PS‐NPs, FFAs, and SR9009. RNA and protein were collected every 4 h from 0 to 48 h for RT‐qPCR and immunoblot analyses, respectively. Treatment‐, time‐, and treatment‐by‐time effects were evaluated as described under Statistical analysis.

### 4.10 Serum Biochemistry

While under isoflurane anesthesia (Solarbio, I8000‐25g), mice underwent cardiac puncture, and the collected blood was transferred into serum‐separation tubes. Blood was allowed to clot for 30 min at room temperature and centrifuged at 5000 × *g* for 10 min. The recovered serum was analyzed on a Hitachi 7180 automated biochemical analyzer (Hitachi High‐Technologies). Internal quality‐control materials were included in every analytical run to monitor assay performance.

### 4.11 Measurement of Hepatic Lipids

Liver portions weighing approximately 50–100 mg were mechanically disrupted in prechilled PBS using a glass homogenizer. After preparation of the lipid‐containing fraction from each homogenate, hepatic TG and T‐CHO were determined with enzymatic colorimetric kits obtained from Nanjing Jiancheng Bioengineering Institute (TG, A110‐1‐1; T‐CHO, A111‐1‐1). Absorbance was measured at 510 nm using a BioTek Synergy H1 microplate reader. Values were normalized to the wet mass of the corresponding liver specimen and reported as mmol/g tissue.

### 4.12 Glucose and Insulin Tolerance Testing

Both tolerance tests were initiated at ZT4. For GTT, mice were fasted for 16 h and given D‐glucose (1 g/kg, i.p.); for ITT, mice were fasted for 4 h and injected with human insulin (0.75 U/kg, i.p.). Tail‐vein glucose was recorded immediately before injection and at 25, 45, 90, and 120 min using an Accu‐Chek Performa glucometer.

### 4.13 Histology and Lipid Staining

Liver samples were fixed in 4% paraformaldehyde (Biosharp, BL539A) at 4°C for 36 h, paraffin‐embedded, sectioned at 5 µm, and stained with hematoxylin and eosin (H&E). For lipid staining, separate samples were embedded in OCT, cut into 10‐µm frozen sections, stained with Oil Red O (Solarbio, G1261‐2) for 15 min, and counterstained with hematoxylin. A blinded pathologist evaluated the NAS score based on steatosis, lobular inflammation, and hepatocellular ballooning [40]. Oil Red O‐positive areas were quantified in ImageJ from at least five random fields per section using a uniform threshold. Field values from each animal were averaged for statistical analysis.

### 4.14 RT‐qPCR

Total RNA was extracted from ∼30 mg liver tissue using Freezol reagent (Vazyme, R711‐01). RNA quality was checked by NanoDrop 2000 and agarose gel electrophoresis. One microgram of RNA was reverse‐transcribed using All‐in‐One Ultra qRT SuperMix (Vazyme, R333‐01). qPCR was performed with ChamQ SYBR qPCR Master Mix (Vazyme, Q311‐02) on a QuantFinder 96 system under the following conditions: 95°C for 30 s, followed by 40 cycles of 95°C for 10 s and 60°C for 30 s. Relative expression was calculated using the 2^^−ΔΔCt^ method with β‐actin as the reference gene. Primer sequences are listed in Table S2.

### 4.15 RNA Sequencing and Bioinformatic Analysis

RNA‐seq was conducted on 40 liver samples. Strand‐specific libraries were prepared from 1 µg total RNA using the Illumina Stranded mRNA Prep kit and sequenced on a DNBSEQ‐T7 platform (PE150). Raw reads were filtered with fastp and aligned to the mouse GRCm39 genome using HISAT2. Gene expression was quantified by RSEM. RSEM counts were used for differential expression analysis, whereas TPM values were used for visualization and sample‐level comparisons.

The dataset comprised 40 libraries, with a median of 43.78 million clean reads per library (range, 39.41–56.26 million). Median overall and unique alignment rates were 96.93% (range, 93.51%–98.92%) and 74.55% (range, 60.99%–85.82%), respectively. All libraries were constructed and sequenced within the same batch; therefore, no batch‐effect correction was applied.

Differential expression was analyzed with DESeq2 using raw count data. GO and KEGG enrichment analyses were performed with GOATOOLS and SciPy, respectively, with Bonferroni correction.

### 4.16 Western Blotting

Liver tissues and treated HepG2/Huh7 cells were lysed in RIPA buffer (Beyotime, P0013B) containing 1 mM PMSF (Beyotime, ST506) and PhosSTOP (Roche, 4906845001). Lysates were centrifuged at 12 000 × *g* for 15 min at 4°C, and protein concentrations were measured by BCA assay (Thermo Fisher Scientific, 23227).

Protein (50 µg) was separated on 8%–12% SDS‐PAGE gels and transferred to 0.22µm PVDF membranes (Millipore, IPVH00010). After blocking with 5% non‐fat milk for 2 h, membranes were incubated overnight at 4°C with antibodies against AMPKα (Affinity, AF6423; 1:2000), p‐AMPKα Thr172 (Affinity, AF3423; 1:2000), BMAL1 (Proteintech, 14268‐1‐AP; 1:1000), or β‐actin (Proteintech, 20536‐1‐AP; 1:5000). HRP‐conjugated secondary antibodies were applied for 1 h. Bands were visualized using ECL (Thermo Fisher Scientific, 32106) on a Tanon 5200 system and quantified in ImageJ. p‐AMPKα was normalized to total AMPKα, while AMPKα and BMAL1 were normalized to β‐actin.

### 4.17 Mitochondrial Membrane Potential

Fresh liver tissues were collected from the same anatomical location within the same hepatic lobe for all experimental groups and processed immediately under ice‐cold conditions. Mitochondria were isolated by differential centrifugation using a commercial mitochondrial isolation kit (Beyotime, C3606, China). The mitochondrial membrane potential (ΔΨm) was assessed with an enhanced JC‐1 detection kit (Beyotime, C2003S, China). Briefly, freshly isolated mitochondrial fractions were incubated with JC‐1 working solution at 37°C for 20 min and then washed twice with the provided staining buffer. All procedures were performed strictly according to the manufacturer's protocol, using only the reagents supplied in the kit without additional supplementation of exogenous respiratory substrates. For cultured hepatocytes, ΔΨm was measured by JC‐1 staining. After treatment, HepG2 and Huh7 cells were incubated with JC‐1 for 20 min at 37°C in the dark, washed twice, and returned to fresh medium.

JC‐1 fluorescence was recorded with a BioTek Synergy H1 reader at 490/530 nm for monomers and 525/590 nm for aggregates. Mitochondrial membrane potential was expressed as the aggregate/monomer fluorescence ratio. To reduce experimental variability, all mitochondrial preparations were analyzed immediately after isolation under identical assay conditions.

Fluorescence images were acquired using a Nikon ECLIPSE Ts2R inverted fluorescence microscope under identical acquisition parameters for all groups. JC‐1 monomers and aggregates were detected in green and red fluorescence channels, respectively. The red‐to‐green fluorescence intensity ratio was quantified from randomly selected fields using ImageJ software and used as an indicator of mitochondrial membrane potential.

### 4.18 ATP and ADP Measurement

ATP and ADP were quantified separately in fresh liver samples and treated hepatocytes. A luciferase‐based ATP kit (Beyotime, S0026, China) was used for ATP detection, whereas ADP was measured with an Amplex Red ADP assay kit (Beyotime, S0307S, China). Liver specimens assigned to the two assays were obtained simultaneously from the same anatomical region but processed as separate aliquots.

For ATP measurement, 20 mg fresh liver was homogenized in chilled ATP lysis buffer and centrifuged at 12 000 × *g* for 5 min at 4°C. Supernatant luminescence was recorded with a GloMax luminometer (Promega, USA). For ADP, a separate liver sample was homogenized in chilled BeyoLy Buffer A, centrifuged at 12 000 × *g* for 5 min at 4°C, and analyzed fluorometrically at 560/590 nm.

HepG2 and Huh7 cells were washed with ice‐cold PBS, lysed with the corresponding extraction buffer, and centrifuged at 12 000 × *g* at 4°C. ATP and ADP concentrations were calculated from standard curves. Liver values were normalized to fresh tissue weight (µmol/g), whereas cellular values were normalized to protein content determined by BCA assay (µmol/g protein). The ATP/ADP ratio was calculated from molar concentrations.

### 4.19 Seahorse Mitochondrial Stress Test

Mitochondrial respiration was measured in HepG2 and Huh7 cells using an Agilent Seahorse XFe96 analyzer. Cells were seeded at 1 × 10^4^ cells/well one day before analysis, with corner wells used for background correction. The sensor cartridge was hydrated overnight at 37°C in a non‐CO_2_ incubator with Seahorse XF calibrant.

Before analysis, cells were equilibrated for 1 h in Seahorse XF DMEM (Agilent, 103575‐100) containing 10 mM glucose, 1 mM sodium pyruvate, and 2 mM glutamine. OCR was recorded before and after sequential addition of oligomycin (1.5 µM), FCCP (1 µM), and rotenone/antimycin A (0.5 µM). Respiratory parameters were calculated using Seahorse Wave v2.4.3. Basal respiration, ATP‐linked respiration, proton leak, and maximal respiration were derived after correction for non‐mitochondrial OCR.

### 4.20 Transmission Electron Microscopy

Immediately after excision, liver tissue was divided into blocks of approximately 1 mm^3^. The specimens were immersed in 2.5% glutaraldehyde at 4°C overnight, rinsed with 0.1 M phosphate buffer, and exposed to 1% osmium tetroxide for 1–2 h at room temperature. Water was removed by passing the samples through progressively higher concentrations of ethanol before epoxy‐resin infiltration and polymerization. Sections approximately 90 nm thick were cut with an ultramicrotome, placed on copper grids, and contrasted sequentially with uranyl acetate and lead citrate before electron‐microscopic examination.

### 4.21 Carnitine Palmitoyltransferase I Activity Assay

CPT1 activity was measured in liver mitochondrial extracts using a colorimetric kit (ml301014; Shanghai Enzyme‐linked Biotechnology, China). The assay detects CPT1‐generated CoA‐SH through its reaction with DTNB to form TNB, measured at 412 nm.

Fresh liver (100 mg) was homogenized on ice in 1 mL extraction buffer A with 10 µL reagent C. After centrifugation at 600 × *g* for 5 min at 4°C, the supernatant was further centrifuged at 11 000 × *g* for 10 min at 4°C. The mitochondrial pellet was resuspended in 200 µL extraction buffer B containing 2 µL reagent C and sonicated for 30 cycles at 20% power (200 W; 3‐s pulse/10‐s interval). Protein concentration was determined by BCA assay.

Each reaction contained 10 µL mitochondrial lysate, 100 µL reagent 1, 100 µL reagent 2, and 30 µL reagent 3. Absorbance at 412 nm was recorded immediately (A_1_) and after 5 min at 37°C (A_2_), with ΔA = A_2_ − A_1_. Activity was calculated using a TNB extinction coefficient of 13 600 L·mol^−^^1^·cm^−^^1^ and a 0.68‐cm path‐length correction, normalized to mitochondrial protein, and expressed as nmol/min/mg protein.

### 4.22 Intracellular ROS Measurement

Intracellular ROS was assessed using H2DCFDA (Biosharp, BL714A). After treatment, cells were incubated with 10 µM H2DCFDA in serum‐free medium for 30 min at 37°C in the dark, washed with PBS, and imaged using an Olympus IX73 fluorescence microscope. Identical imaging settings were applied to all groups. Six random fields were analyzed per independent culture in ImageJ, and the mean fluorescence intensity was averaged to obtain one value for each biological replicate.

### 4.23 Statistical Analysis

Unless otherwise specified, numerical results are reported as the mean ± SD. Independent animals or independently prepared cell cultures were regarded as biological replicates and constituted the units used for statistical testing. Normality and equality of variance were examined using the Shapiro–Wilk and Levene tests, respectively.

Datasets containing one experimental factor were evaluated by one‐way analysis of variance (ANOVA). For factorial experiments, two‐way ANOVA was applied, with diet or FFA treatment and PS‐NPs exposure or concentration entered as the two factors. Where appropriate, pairwise group differences were examined using Tukey‐adjusted comparisons.Circadian time‐course experiments involved separate cultures collected at each sampling point and were therefore evaluated by two‐way ANOVA incorporating treatment, sampling time, and the treatment × time interaction. The corresponding *F* and *p* values for the main and interaction effects are provided in Table S3.

Animal experiments included six mice in each group. Group allocation was randomized, and histopathological assessment, image quantification, and downstream data analysis were conducted without knowledge of treatment assignment. All statistical tests were two‐sided, and *p* < 0.05 was considered statistically significant.

## Author Contributions

Y.S. and Z.D. conceptualized the study and developed the experimental design. P.X., S.H., and Y.S. carried out the experimental procedures. P.X. and S.H. performed data processing and statistical analyses. L.Z., J.H., K.L., G.L., J.W., M.Z., and F.Z. contributed to methodological development, experimental implementation, and technical support. Y.S. supervised the research project and secured financial support. P.X. and Y.S. prepared the initial manuscript draft. All authors participated in data interpretation, provided critical revisions, and approved the final manuscript.

## Funding

This research was supported by the Zhejiang Provincial Natural Science Foundation of China under Grant No. LQN26B070007 and the Research Project of Zhejiang Chinese Medical University under Grant No. 2024RCZXZK40.

## Conflicts of Interest

The authors declare no conflicts of interest.

## Supporting information




**Supporting File 1**: advs77840‐sup‐0001‐SuppMat.docx.

**Supporting File 2**: advs77840‐sup‐0002‐SuppMat.docx.

## Data Availability

The data that support the findings of this study are available from the corresponding author upon reasonable request.
