# Supplementary material for: Developmental Environmental Chronotoxicity by Polystyrene Nanoplastics Exacerbates High‐Fat Diet‐Induced Pediatric MAFLD Through Circadian Disruption and Mitochondrial Dysfunction
Source: Adv Sci (Weinh). 2026 Sep 27:e77840. Online ahead of print. doi: 10.1002/advs.77840 (PMC13616410; doi:10.1002/advs.77840)
Supplement: Supplementary file 1 [file ADVS-9999-e77840-s001.docx]

**Supplementary Table 1. Estimated weekly PS-NPs exposure (μg/g body weight/week) during the first eight weeks**

| **Age** | **Group** | **Week 1** | **Week 2** | **Week 3** | **Week 4** | **Week 5** | **Week 6** | **Week 7** | **Week 8** | **average** | **8-week cumulative (μg/g body weight)** |
| --- | --- | --- | --- | --- | --- | --- | --- | --- | --- | --- | --- |
| 3w | PS | 26.18 | 24.30 | 24.59 | 29.37 | 25.50 | 25.06 | 23.64 | 25.61 | 25.53 | 204.24 |
| 3w | HP | 24.21 | 20.00 | 19.12 | 20.34 | 19.64 | 28.57 | 22.82 | 18.86 | 21.69 | 173.56 |
| 8w | PS | 23.79 | 24.64 | 23.01 | 26.63 | 26.63 | 21.47 | 24.67 | 22.50 | 24.17 | 193.33 |
| 8w | HP | 25.22 | 20.42 | 24.45 | 25.82 | 26.04 | 25.10 | 25.06 | 26.35 | 24.81 | 198.46 |

**Supplementary Table 2. Primer sequences used for RT-qPCR**

| **Primer** | **Sequence (5’-3’)** |
| --- | --- |
| mouse Bmal1-F | ACAGTCAGATTGAAAAGAGGCG |
| mouse Bmal1-R | GCCATCCTTAGCACGGTGAG |
| mouse Clock-F | CTTCCTGGTAACGCGAGAAAG |
| mouse Clock-R | GTCGAATCTCACTAGCATCTGAC |
| mouse Per2-F | CTCCAGCGGAAACGAGAACTG |
| mouse Per2-R | TTGGCAGACTGCTCACTACTG |
| mouse Srebp1c-F | GAGCCATGGATTGCACATTT |
| mouse Srebp1c-R | CTCAGGAGAGTTGGCACCTG |
| mouse LXRα-F | TGGAGAACTCAAAGATGGGG |
| mouse LXRα-R | TGAGAGCATCACCTTCCTCA |
| mouse Fas-F | GGAGGTGGTGATAGCCGGTAT |
| mouse Fas-R | TGGGTAATCCATAGAGCCCAG |
| mouse Acadl-F | TGCCCTATATTGCGAATTACGG |
| mouse Acadl-R | CTATGGCACCGATACACTTGC |
| mouse PGC-1α-F | TATGGAGTGACATAGAGTGTGCT |
| mouse PGC-1α-R | GTCGCTACACCACTTCAATCC |
| mouse Cpt1a-F | AGATCAATCGGACCCTAGACAC |
| mouse Cpt1a-R | CAGCGAGTAGCGCATAGTCA |
| mouse SDHA-F | GGAACACTCCAAAAACAGACCT |
| mouse SDHA-R | CCACCACTGGGTATTGAGTAGAA |
| mouse Ndufa9-F | GTCCGCTTTCGGGTTGTTAGA |
| mouse Ndufa9-R | CCTCCTTTCCCGTGAGGTA |
| mouse ATP5A1-F | TCTCCATGCCTCTAACACTCG |
| mouse ATP5A1 -R | CCAGGTCAACAGACGTGTCAG |
| human Fas-F | TCTGGTTCTTACGTCTGTTGC |
| human FAS-R | CTGTGCAGTCCCTAGCTTTCC |
| human LXRα-F | CCTTCAGAACCCACAGAGATCC |
| human LXRα-R | ACGCTGCATAGCTCGTTCC |
| human Bmal1-F | AAGGGAAGCTCACAGTCAGAT |
| human Bmal1-R | GGACATTGCGTTGCATGTTGG |
| human ACADL-F | AGGGGATCTGTACTCCGCAG |
| human ACADL-R | CTCTGTCATTGCTATTGCACCA |
| human clock-F | TGCGAGGAACAATAGACCCAA |
| human clock-R | ATGGCCATGTGTGCGTTGTA |
| human PGC-1α-F | TCTGAGTCTGTATGGAGTGACAT |
| human PGC-1α-R | CCAAGTCGTTCACATCTAGTTCA |
| human ATP5a1-F | GTATTGCCCGCGTACATGG |
| human ATP5A1-R | AGGACATACCCTTTAAGCCTGA |
| human NDUFA9-F | GTCACGTTCTGCCATTACTGC |
| human NDUFA9-R | GGTGGTTGACAACATATCGCC |
| human β-actin-F | CATGTACGTTGCTATCCAGGC |
| human β-actin-R | CTCCTTAATGTCACGCACGAT |

**Supplementary Table 2. Primer sequences used for RT-qPCR (continued)**

| **Primer** | **Sequence (5’-3’)** |
| --- | --- |
| mouse β-actin-F | GGCTGTATTCCCCTCCATCG |
| mouse β-actin-R | CCAGTTGGTAACAATGCCATGT |

**Supplementary Table 3. Two-way ANOVA results**

**A. Animal experiments: HFD × PS-NPs**

| **Figure panel and outcome** | **Age** | **HFD effect** | **PS-NPs effect** | **HFD × PS-NPs interaction** |
| --- | --- | --- | --- | --- |
| Fig. 1b, Liver index | 3w | F(1, 20) = 52.92; *p*<0.0001 | F(1, 20) = 3.11; *p*=0.0930 | F(1, 20) = 7.45; *p*=0.0129 |
| Fig. 1b, Liver index | 8w | F(1, 20) = 27.82; *p*<0.0001 | F(1, 20) = 0.77; *p*=0.3911 | F(1, 20) = 1.44; *p*=0.2444 |
| Fig. 1c, ALT | 3w | F(1, 20) = 30.91; *p*<0.0001 | F(1, 20) = 23.93; *p*<0.0001 | F(1, 20) = 0.69; *p*=0.4169 |
| Fig. 1c, ALT | 8w | F(1, 20) = 8.17; *p*=0.0097 | F(1, 20) = 1.64; *p*=0.2144 | F(1, 20) = 3.76; *p*=0.0667 |
| Fig. 1c, AST | 3w | F(1, 20) = 30.78; *p*<0.0001 | F(1, 20) = 15.72; *p*=0.0008 | F(1, 20) = 0.84; *p*=0.3715 |
| Fig. 1c, AST | 8w | F(1, 20) = 11.60; *p*=0.0028 | F(1, 20) = 0.95; *p*=0.3422 | F(1, 20) = 2.06; *p*=0.1668 |
| Fig. 1d, LDL-C | 3w | F(1, 20) = 23.54; *p*<0.0001 | F(1, 20) = 15.58; *p*=0.0008 | F(1, 20) = 0.17; *p*=0.6853 |
| Fig. 1d, LDL-C | 8w | F(1, 20) = 18.86; *p*=0.0003 | F(1, 20) = 3.63; *p*=0.0712 | F(1, 20) = 3.63; *p*=0.0712 |
| Fig. 1d, HDL-C | 3w | F(1, 20) = 19.04; *p*=0.0003 | F(1, 20) = 16.67; *p*=0.0006 | F(1, 20) = 9.67; *p*=0.0055 |
| Fig. 1d, HDL-C | 8w | F(1, 20) = 27.44; *p*<0.0001 | F(1, 20) = 0.05; *p*=0.8294 | F(1, 20) = 0.89; *p*=0.3556 |
| Fig. 1e, Serum TG | 3w | F(1, 20) = 28.56; *p*<0.0001 | F(1, 20) = 15.15; *p*=0.0009 | F(1, 20) = 17.09; *p*=0.0005 |
| Fig. 1e, Serum TG | 8w | F(1, 20) = 17.29; *p*=0.0005 | F(1, 20) = 2.06; *p*=0.1667 | F(1, 20) = 0.00; *p*=0.9769 |
| Fig. 1e, Serum T-CHO | 3w | F(1, 20) = 91.20; *p*<0.0001 | F(1, 20) = 31.95; *p*<0.0001 | F(1, 20) = 0.19; *p*=0.6644 |
| Fig. 1e, Serum T-CHO | 8w | F(1, 20) = 173.85; *p*<0.0001 | F(1, 20) = 0.60; *p*=0.4465 | F(1, 20) = 12.03; *p*=0.0024 |
| Fig. 1f, Hepatic TG | 3w | F(1, 20) = 86.35; *p*<0.0001 | F(1, 20) = 12.86; *p*=0.0018 | F(1, 20) = 4.37; *p*=0.0494 |
| Fig. 1f, Hepatic TG | 8w | F(1, 20) = 53.90; *p*<0.0001 | F(1, 20) = 0.00; *p*=0.9561 | F(1, 20) = 1.49; *p*=0.2365 |
| Fig. 1f, Hepatic T-CHO | 3w | F(1, 20) = 181.99; *p*<0.0001 | F(1, 20) = 32.57; *p*<0.0001 | F(1, 20) = 1.15; *p*=0.2965 |
| Fig. 1f, Hepatic T-CHO | 8w | F(1, 20) = 52.69; *p*<0.0001 | F(1, 20) = 4.99; *p*=0.0370 | F(1, 20) = 2.22; *p*=0.1523 |
| Fig. 1g, NAS score | 3w | F(1, 20) = 37.04; *p*<0.0001 | F(1, 20) = 9.26; *p*=0.0064 | F(1, 20) = 3.33; *p*=0.0829 |
| Fig. 1g, NAS score | 8w | F(1, 20) = 0.00; *p*=1.0000 | F(1, 20) = 0.26; *p*=0.6181 | F(1, 20) = 0.26; *p*=0.6181 |
| Fig. 1h, Oil Red O-positive area | 3w | F(1, 20) = 670.07; *p*<0.0001 | F(1, 20) = 350.14; *p*<0.0001 | F(1, 20) = 5.01; *p*=0.0368 |
| Fig. 1h, Oil Red O-positive area | 8w | F(1, 20) = 498.93; *p*<0.0001 | F(1, 20) = 47.34; *p*<0.0001 | F(1, 20) = 33.39; *p*<0.0001 |
| Fig. 4a, BMAL1 protein | 3w | F(1, 16) = 2.50; *p*=0.1334 | F(1, 16) = 1.68; *p*=0.2136 | F(1, 16) = 40.55; *p*<0.0001 |
| Fig. 4a, BMAL1 protein | 8w | F(1, 16) = 1.25; *p*=0.2802 | F(1, 16) = 0.02; *p*=0.9030 | F(1, 16) = 0.05; *p*=0.8305 |

**A. Animal experiments: HFD × PS-NPs (continued)**

| **Figure panel and outcome** | **Age** | **HFD effect** | **PS-NPs effect** | **HFD × PS-NPs interaction** |
| --- | --- | --- | --- | --- |
| Fig. 4b, p-AMPK/t-AMPK ratio | 3w | F(1, 16) = 36.59; *p*<0.0001 | F(1, 16) = 25.33; *p*=0.0001 | F(1, 16) = 0.74; *p*=0.4011 |
| Fig. 4b, p-AMPK/t-AMPK ratio | 8w | F(1, 16) = 0.05; *p*=0.8216 | F(1, 16) = 0.03; *p*=0.8709 | F(1, 16) = 7.12; *p*=0.0168 |
| Fig. 4c, ATP/ADP ratio | 3w | F(1, 20) = 16.40; *p*=0.0006 | F(1, 20) = 19.52; *p*=0.0003 | F(1, 20) = 2.77; *p*=0.1114 |
| Fig. 4c, ATP/ADP ratio | 8w | F(1, 20) = 1.04; *p*=0.3195 | F(1, 20) = 0.98; *p*=0.3345 | F(1, 20) = 0.00; *p*=0.9971 |
| Fig. 4d, CPT1 activity | 3w | F(1, 20) = 20.48; *p*=0.0002 | F(1, 20) = 21.48; *p*=0.0002 | F(1, 20) = 0.02; *p*=0.8854 |
| Fig. 4d, CPT1 activity | 8w | F(1, 20) = 0.49; *p*=0.4906 | F(1, 20) = 3.70; *p*=0.0686 | F(1, 20) = 1.18; *p*=0.2898 |
| Fig. 4e, Mitochondrial area | 3w | F(1, 96) = 26.23; *p*<0.0001 | F(1, 96) = 0.62; *p*=0.4312 | F(1, 96) = 22.11; *p*<0.0001 |
| Fig. 4e, Mitochondrial area | 8w | F(1, 96) = 1.64; *p*=0.2032 | F(1, 96) = 1.05; *p*=0.3092 | F(1, 96) = 0.09; *p*=0.7657 |
| Fig. 4e, Cristae density | 3w | F(1, 96) = 11.72; *p*=0.0009 | F(1, 96) = 15.63; *p*=0.0001 | F(1, 96) = 0.04; *p*=0.8354 |
| Fig. 4e, Cristae density | 8w | F(1, 96) = 1.84; *p*=0.1780 | F(1, 96) = 3.34; *p*=0.0709 | F(1, 96) = 3.36; *p*=0.0698 |
| Fig. 4f, Hepatic ATP content | 3w | F(1, 20) = 85.90; *p*<0.0001 | F(1, 20) = 19.20; *p*=0.0003 | F(1, 20) = 0.17; *p*=0.6855 |
| Fig. 4f, Hepatic ATP content | 8w | F(1, 20) = 0.07; *p*=0.7960 | F(1, 20) = 0.18; *p*=0.6719 | F(1, 20) = 0.19; *p*=0.6648 |
| Fig. 4g, Mitochondrial membrane potential | 3w | F(1, 20) = 30.76; *p*<0.0001 | F(1, 20) = 25.87; *p*<0.0001 | F(1, 20) = 0.04; *p*=0.8481 |
| Fig. 4g, Mitochondrial membrane potential | 8w | F(1, 20) = 0.03; *p*=0.8725 | F(1, 20) = 8.67; *p*=0.0080 | F(1, 20) = 0.50; *p*=0.4879 |
| Fig. 4h, Bmal1 | 3w | F(1, 20) = 43.57; *p*<0.0001 | F(1, 20) = 20.94; *p*=0.0002 | F(1, 20) = 140.65; *p*<0.0001 |
| Fig. 4h, Bmal1 | 8w | F(1, 20) = 0.00; *p*=0.9512 | F(1, 20) = 0.18; *p*=0.6734 | F(1, 20) = 0.08; *p*=0.7865 |
| Fig. 4h, Clock | 3w | F(1, 20) = 9.34; *p*=0.0062 | F(1, 20) = 11.23; *p*=0.0032 | F(1, 20) = 68.81; *p*<0.0001 |
| Fig. 4h, Clock | 8w | F(1, 20) = 7.35; *p*=0.0134 | F(1, 20) = 4.12; *p*=0.0558 | F(1, 20) = 0.47; *p*=0.4995 |
| Fig. 4h, Npas2 | 3w | F(1, 20) = 8.59; *p*=0.0083 | F(1, 20) = 9.36; *p*=0.0062 | F(1, 20) = 24.07; *p*<0.0001 |
| Fig. 4h, Npas2 | 8w | F(1, 20) = 2.42; *p*=0.1351 | F(1, 20) = 1.46; *p*=0.2417 | F(1, 20) = 7.49; *p*=0.0127 |
| Fig. 4h, Fas | 3w | F(1, 20) = 36.87; *p*<0.0001 | F(1, 20) = 27.64; *p*<0.0001 | F(1, 20) = 0.25; *p*=0.6248 |
| Fig. 4h, Fas | 8w | F(1, 20) = 0.12; *p*=0.7331 | F(1, 20) = 0.60; *p*=0.4479 | F(1, 20) = 0.17; *p*=0.6859 |
| Fig. 4h, LXR | 3w | F(1, 20) = 26.70; *p*<0.0001 | F(1, 20) = 23.85; *p*<0.0001 | F(1, 20) = 0.06; *p*=0.8135 |
| Fig. 4h, LXR | 8w | F(1, 20) = 4.36; *p*=0.0497 | F(1, 20) = 0.31; *p*=0.5847 | F(1, 20) = 0.21; *p*=0.6505 |
| Fig. 4h, Srebp1c | 3w | F(1, 20) = 85.17; *p*<0.0001 | F(1, 20) = 28.52; *p*<0.0001 | F(1, 20) = 0.27; *p*=0.6109 |
| Fig. 4h, Srebp1c | 8w | F(1, 20) = 0.20; *p*=0.6626 | F(1, 20) = 3.69; *p*=0.0691 | F(1, 20) = 1.89; *p*=0.1839 |
| Fig. 4h, Acadl | 3w | F(1, 20) = 0.10; *p*=0.7552 | F(1, 20) = 9.25; *p*=0.0064 | F(1, 20) = 58.03; *p*<0.0001 |
| Fig. 4h, Acadl | 8w | F(1, 20) = 4.29; *p*=0.0514 | F(1, 20) = 0.27; *p*=0.6110 | F(1, 20) = 0.57; *p*=0.4585 |

**A. Animal experiments: HFD × PS-NPs (continued)**

| **Figure panel and outcome** | **Age** | **HFD effect** | **PS-NPs effect** | **HFD × PS-NPs interaction** |
| --- | --- | --- | --- | --- |
| Fig. 4h, Pgc-1α | 8w | F(1, 20) = 3.38; *p*=0.0810 | F(1, 20) = 0.07; *p*=0.7935 | F(1, 20) = 0.32; *p*=0.5758 |
| Fig. 4h, Cpt1a | 3w | F(1, 20) = 2.78; *p*=0.1111 | F(1, 20) = 23.30; *p*=0.0001 | F(1, 20) = 84.75; *p*<0.0001 |
| Fig. 4h, Cpt1a | 8w | F(1, 20) = 0.94; *p*=0.3427 | F(1, 20) = 0.25; *p*=0.6240 | F(1, 20) = 4.51; *p*=0.0464 |
| Fig. 4h, Atp5a1 | 3w | F(1, 20) = 11.62; *p*=0.0028 | F(1, 20) = 0.07; *p*=0.8006 | F(1, 20) = 52.84; *p*<0.0001 |
| Fig. 4h, Atp5a1 | 8w | F(1, 20) = 3.40; *p*=0.0799 | F(1, 20) = 10.48; *p*=0.0041 | F(1, 20) = 0.01; *p*=0.9144 |
| Fig. 4h, Ndufa9 | 3w | F(1, 20) = 106.66; *p*<0.0001 | F(1, 20) = 4.04; *p*=0.0580 | F(1, 20) = 22.52; *p*=0.0001 |
| Fig. 4h, Ndufa9 | 8w | F(1, 20) = 10.04; *p*=0.0048 | F(1, 20) = 5.77; *p*=0.0262 | F(1, 20) = 1.57; *p*=0.2240 |
| Fig. 4h, Sdha | 3w | F(1, 20) = 8.44; *p*=0.0087 | F(1, 20) = 2.49; *p*=0.1304 | F(1, 20) = 33.99; *p*<0.0001 |
| Fig. 4h, Sdha | 8w | F(1, 20) = 0.18; *p*=0.6717 | F(1, 20) = 16.64; *p*=0.0006 | F(1, 20) = 0.31; *p*=0.5816 |
| Supplementary Fig. 1a, Body weight-normalized water intake | 3w | F(1, 28) = 17.08; *p*=0.0003 | F(1, 28) = 1.79; *p*=0.1917 | F(1, 28) = 0.01; *p*=0.9172 |
| Supplementary Fig. 1a, Body weight-normalized water intake | 8w | F(1, 28) = 0.53; *p*=0.4722 | F(1, 28) = 2.69; *p*=0.1121 | F(1, 28) = 0.01; *p*=0.9270 |
| Supplementary Fig. 1b, Body weight-normalized food intake | 3w | F(1, 28) = 0.10; *p*=0.7561 | F(1, 28) = 0.01; *p*=0.9233 | F(1, 28) = 1.39; *p*=0.2482 |
| Supplementary Fig. 1b, Body weight-normalized food intake | 8w | F(1, 28) = 0.10; *p*=0.7582 | F(1, 28) = 0.48; *p*=0.4941 | F(1, 28) = 1.01; *p*=0.3234 |
| Supplementary Fig. 1d, Body weight-normalized energy intake | 3w | F(1, 28) = 539.16; *p*<0.0001 | F(1, 28) = 0.12; *p*=0.7277 | F(1, 28) = 1.25; *p*=0.2739 |
| Supplementary Fig. 1d, Body weight-normalized energy intake | 8w | F(1, 28) = 564.60; *p*<0.0001 | F(1, 28) = 0.81; *p*=0.3762 | F(1, 28) = 1.27; *p*=0.2688 |
| Supplementary Fig. 3a, Body weight gain | 3w | F(1, 20) = 46.57; *p*<0.0001 | F(1, 20) = 0.54; *p*=0.4713 | F(1, 20) = 2.51; *p*=0.1290 |
| Supplementary Fig. 3a, Body weight gain | 8w | F(1, 20) = 66.28; *p*<0.0001 | F(1, 20) = 0.10; *p*=0.7543 | F(1, 20) = 0.02; *p*=0.9007 |
| Supplementary Fig. 3b, Total fat index | 3w | F(1, 20) = 205.23; *p*<0.0001 | F(1, 20) = 4.09; *p*=0.0567 | F(1, 20) = 0.08; *p*=0.7750 |
| Supplementary Fig. 3b, Total fat index | 8w | F(1, 20) = 52.62; *p*<0.0001 | F(1, 20) = 4.75; *p*=0.0415 | F(1, 20) = 1.61; *p*=0.2191 |

**B. Cell experiments: FFA × PS-NPs dose**

| **Figure panel and outcome** | **Cell line** | **FFA effect** | **PS-NPs dose effect** | **FFA × PS-NPs interaction** |
| --- | --- | --- | --- | --- |
| Fig. 5a, Oil Red O-positive area | HepG2 | F(1, 30) = 151.12; *p*<0.0001 | F(2, 30) = 45.58; *p*<0.0001 | F(2, 30) = 4.07; *p*=0.0272 |
| Fig. 5a, Oil Red O-positive area | Huh7 | F(1, 30) = 319.28; *p*<0.0001 | F(2, 30) = 32.38; *p*<0.0001 | F(2, 30) = 15.87; *p*<0.0001 |
| Fig. 5b, Intracellular TG | HepG2 | F(1, 30) = 51.69; *p*<0.0001 | F(2, 30) = 12.83; *p*<0.0001 | F(2, 30) = 1.09; *p*=0.3478 |
| Fig. 5b, Intracellular TG | Huh7 | F(1, 30) = 25.38; *p*<0.0001 | F(2, 30) = 16.00; *p*<0.0001 | F(2, 30) = 0.95; *p*=0.3974 |

**B. Cell experiments: FFA × PS-NPs dose (continued)**

| **Figure panel and outcome** | **Cell line** | **FFA effect** | **PS-NPs dose effect** | **FFA × PS-NPs interaction** |
| --- | --- | --- | --- | --- |
| Fig. 5c, BMAL1 protein | HepG2 | F(1, 12) = 421.00; *p*<0.0001 | F(2, 12) = 39.75; *p*<0.0001 | F(2, 12) = 3.61; *p*=0.0591 |
| Fig. 5c, p-AMPK/t-AMPK ratio | HepG2 | F(1, 12) = 96.11; *p*<0.0001 | F(2, 12) = 25.73; *p*<0.0001 | F(2, 12) = 0.98; *p*=0.4050 |
| Fig. 5c, p-AMPK/t-AMPK ratio | Huh7 | F(1, 12) = 161.64; *p*<0.0001 | F(2, 12) = 18.74; *p*=0.0002 | F(2, 12) = 4.80; *p*=0.0294 |
| Fig. 5f, BMAL1 | HepG2 | F(1, 30) = 26.98; *p*<0.0001 | F(2, 30) = 20.44; *p*<0.0001 | F(2, 30) = 2.65; *p*=0.0870 |
| Fig. 5f, CLOCK | HepG2 | F(1, 30) = 64.72; *p*<0.0001 | F(2, 30) = 35.47; *p*<0.0001 | F(2, 30) = 2.30; *p*=0.1181 |
| Fig. 5f, FAS | HepG2 | F(1, 30) = 61.63; *p*<0.0001 | F(2, 30) = 11.49; *p*=0.0002 | F(2, 30) = 1.66; *p*=0.2079 |
| Fig. 5f, LXRα | HepG2 | F(1, 30) = 23.16; *p*<0.0001 | F(2, 30) = 31.01; *p*<0.0001 | F(2, 30) = 6.84; *p*=0.0036 |
| Fig. 5f, BMAL1 | Huh7 | F(1, 30) = 53.82; *p*<0.0001 | F(2, 30) = 24.76; *p*<0.0001 | F(2, 30) = 0.12; *p*=0.8900 |
| Fig. 5f, CLOCK | Huh7 | F(1, 30) = 40.44; *p*<0.0001 | F(2, 30) = 36.92; *p*<0.0001 | F(2, 30) = 0.13; *p*=0.8775 |
| Fig. 5f, FAS | Huh7 | F(1, 30) = 146.13; *p*<0.0001 | F(2, 30) = 56.93; *p*<0.0001 | F(2, 30) = 13.17; *p*<0.0001 |
| Fig. 5f, LXRα | Huh7 | F(1, 30) = 22.63; *p*<0.0001 | F(2, 30) = 21.13; *p*<0.0001 | F(2, 30) = 0.17; *p*=0.8411 |
| Fig. 6a, JC-1 red/green ratio | HepG2 | F(1, 30) = 17.17; *p*=0.0003 | F(2, 30) = 33.63; *p*<0.0001 | F(2, 30) = 1.47; *p*=0.2465 |
| Fig. 6a, JC-1 red/green ratio | Huh7 | F(1, 30) = 21.84; *p*<0.0001 | F(2, 30) = 25.28; *p*<0.0001 | F(2, 30) = 1.13; *p*=0.3367 |
| Fig. 6b, ATP level | HepG2 | F(1, 30) = 58.95; *p*<0.0001 | F(2, 30) = 17.76; *p*<0.0001 | F(2, 30) = 0.18; *p*=0.8350 |
| Fig. 6b, ATP level | Huh7 | F(1, 30) = 19.34; *p*=0.0001 | F(2, 30) = 16.64; *p*<0.0001 | F(2, 30) = 1.63; *p*=0.2132 |
| Fig. 6d, ATP-linked OCR | HepG2 | F(1, 30) = 178.26; *p*<0.0001 | F(2, 30) = 36.05; *p*<0.0001 | F(2, 30) = 1.67; *p*=0.2047 |
| Fig. 6d, ATP-linked OCR | Huh7 | F(1, 30) = 32.53; *p*<0.0001 | F(2, 30) = 31.97; *p*<0.0001 | F(2, 30) = 3.51; *p*=0.0426 |
| Fig. 6d, Proton leak OCR | HepG2 | F(1, 30) = 71.39; *p*<0.0001 | F(2, 30) = 40.46; *p*<0.0001 | F(2, 30) = 12.56; *p*=0.0001 |
| Fig. 6d, Proton leak OCR | Huh7 | F(1, 30) = 44.88; *p*<0.0001 | F(2, 30) = 21.97; *p*<0.0001 | F(2, 30) = 1.09; *p*=0.3478 |
| Fig. 6d, Maximal mitochondrial respiration | HepG2 | F(1, 30) = 176.32; *p*<0.0001 | F(2, 30) = 45.93; *p*<0.0001 | F(2, 30) = 2.17; *p*=0.1322 |
| Fig. 6d, Maximal mitochondrial respiration | Huh7 | F(1, 30) = 62.79; *p*<0.0001 | F(2, 30) = 43.43; *p*<0.0001 | F(2, 30) = 0.62; *p*=0.5430 |
| Fig. 6c, ROS level | HepG2 | F(1, 30) = 86.89; *p*<0.0001 | F(2, 30) = 9.68; *p*=0.0006 | F(2, 30) = 1.56; *p*=0.2270 |
| Fig. 6c, ROS level | Huh7 | F(1, 30) = 97.90; *p*<0.0001 | F(2, 30) = 37.37; *p*<0.0001 | F(2, 30) = 0.44; *p*=0.6453 |
| Fig. 6e, ACADL | HepG2 | F(1, 30) = 88.23; *p*<0.0001 | F(2, 30) = 26.34; *p*<0.0001 | F(2, 30) = 0.90; *p*=0.4186 |
| Fig. 6e, PGC-1α | HepG2 | F(1, 30) = 51.16; *p*<0.0001 | F(2, 30) = 20.76; *p*<0.0001 | F(2, 30) = 0.20; *p*=0.8224 |
| Fig. 6e, NDUFA9 | HepG2 | F(1, 30) = 71.32; *p*<0.0001 | F(2, 30) = 30.38; *p*<0.0001 | F(2, 30) = 2.03; *p*=0.1492 |
| Fig. 6e, ATP5A1 | HepG2 | F(1, 30) = 8.20; *p*=0.0076 | F(2, 30) = 19.07; *p*<0.0001 | F(2, 30) = 2.87; *p*=0.0723 |
| Fig. 6e, ACADL | Huh7 | F(1, 30) = 20.53; *p*<0.0001 | F(2, 30) = 26.59; *p*<0.0001 | F(2, 30) = 0.58; *p*=0.5656 |

**B. Cell experiments: FFA × PS-NPs dose (continued)**

| **Figure panel and outcome** | **Cell line** | **FFA effect** | **PS-NPs dose effect** | **FFA × PS-NPs interaction** |
| --- | --- | --- | --- | --- |
| Fig. 6e, PGC-1α | Huh7 | F(1, 30) = 59.14; *p*<0.0001 | F(2, 30) = 21.69; *p*<0.0001 | F(2, 30) = 5.16; *p*=0.0118 |
| Fig. 6e, NDUFA9 | Huh7 | F(1, 30) = 113.77; *p*<0.0001 | F(2, 30) = 9.66; *p*=0.0006 | F(2, 30) = 0.03; *p*=0.9740 |
| Fig. 6e, ATP5A1 | Huh7 | F(1, 30) = 68.43; *p*<0.0001 | F(2, 30) = 18.31; *p*<0.0001 | F(2, 30) = 2.75; *p*=0.0802 |

**C. Time-course experiments: treatment × time**

| **Figure panel and outcome** | **Stratum** | **Treatment effect** | **Time effect** | **Treatment × time interaction** |
| --- | --- | --- | --- | --- |
| Fig. 5d, BMAL1 protein time course | HepG2 | F(5, 156) = 74.71; *p*<0.0001 | F(12, 156) = 40.52; *p*<0.0001 | F(60, 156) = 7.20; *p*<0.0001 |
| Fig. 5d, BMAL1 protein time course | Huh7 | F(5, 156) = 47.66; *p*<0.0001 | F(12, 156) = 15.88; *p*<0.0001 | F(60, 156) = 10.03; *p*<0.0001 |
| Fig. 5e, Bmal1 mRNA time course | HepG2 | F(5, 390) = 85.57; *p*<0.0001 | F(12, 390) = 28.49; *p*<0.0001 | F(60, 390) = 32.42; *p*<0.0001 |
| Fig. 5e, Bmal1 mRNA time course | Huh7 | F(5, 390) = 27.02; *p*<0.0001 | F(12, 390) = 9.92; *p*<0.0001 | F(60, 390) = 7.54; *p*<0.0001 |
| Fig. 8b, BMAL1 protein time course | HepG2 | F(2, 78) = 924.91; *p*<0.0001 | F(12, 78) = 67.63; *p*<0.0001 | F(24, 78) = 32.70; *p*<0.0001 |
| Fig. 8b, BMAL1 protein time course | Huh7 | F(2, 78) = 347.88; *p*<0.0001 | F(12, 78) = 36.62; *p*<0.0001 | F(24, 78) = 22.56; *p*<0.0001 |
| Fig. 8c, Bmal1 mRNA time course | HepG2 | F(2, 195) = 183.68; *p*<0.0001 | F(12, 195) = 72.94; *p*<0.0001 | F(24, 195) = 44.51; *p*<0.0001 |
| Fig. 8c, Bmal1 mRNA time course | Huh7 | F(2, 195) = 48.94; *p*<0.0001 | F(12, 195) = 7.87; *p*<0.0001 | F(24, 195) = 11.34; *p*<0.0001 |

**D. GTT and ITT per-time-point analyses: HFD × PS-NPs**

| **Outcome** | **Age/time (min)** | **HFD effect** | **PS-NPs effect** | **HFD × PS-NPs interaction** |
| --- | --- | --- | --- | --- |
| GTT | 3w/0 | F(1, 20) = 1.66; *p*=0.2129 | F(1, 20) = 0.33; *p*=0.5738 | F(1, 20) = 4.20; *p*=0.0538 |
| GTT | 3w/25 | F(1, 20) = 19.95; *p*=0.0002 | F(1, 20) = 1.91; *p*=0.1818 | F(1, 20) = 2.54; *p*=0.1265 |
| GTT | 3w/45 | F(1, 20) = 30.63; *p*<0.0001 | F(1, 20) = 1.32; *p*=0.2633 | F(1, 20) = 0.06; *p*=0.8050 |
| GTT | 3w/90 | F(1, 20) = 20.40; *p*=0.0002 | F(1, 20) = 1.46; *p*=0.2415 | F(1, 20) = 0.26; *p*=0.6182 |
| GTT | 3w/120 | F(1, 20) = 27.85; *p*<0.0001 | F(1, 20) = 0.32; *p*=0.5757 | F(1, 20) = 0.00; *p*=0.9845 |
| GTT | 8w/0 | F(1, 20) = 6.75; *p*=0.0172 | F(1, 20) = 8.01; *p*=0.0103 | F(1, 20) = 1.46; *p*=0.2418 |
| GTT | 8w/25 | F(1, 20) = 16.16; *p*=0.0007 | F(1, 20) = 0.14; *p*=0.7108 | F(1, 20) = 0.07; *p*=0.7982 |
| GTT | 8w/45 | F(1, 20) = 56.77; *p*<0.0001 | F(1, 20) = 0.34; *p*=0.5687 | F(1, 20) = 0.90; *p*=0.3549 |
| GTT | 8w/90 | F(1, 20) = 53.49; *p*<0.0001 | F(1, 20) = 3.70; *p*=0.0686 | F(1, 20) = 3.26; *p*=0.0860 |
| GTT | 8w/120 | F(1, 20) = 13.56; *p*=0.0015 | F(1, 20) = 1.00; *p*=0.3295 | F(1, 20) = 3.20; *p*=0.0888 |
| ITT | 3w/0 | F(1, 20) = 0.57; *p*=0.4598 | F(1, 20) = 0.46; *p*=0.5065 | F(1, 20) = 0.11; *p*=0.7459 |
| ITT | 3w/25 | F(1, 20) = 0.30; *p*=0.5904 | F(1, 20) = 2.31; *p*=0.1442 | F(1, 20) = 0.00; *p*=0.9521 |
| ITT | 3w/45 | F(1, 20) = 0.48; *p*=0.4962 | F(1, 20) = 0.15; *p*=0.7042 | F(1, 20) = 0.00; *p*=0.9879 |
| ITT | 3w/90 | F(1, 20) = 0.24; *p*=0.6330 | F(1, 20) = 4.32; *p*=0.0507 | F(1, 20) = 1.83; *p*=0.1918 |
| ITT | 3w/120 | F(1, 20) = 0.13; *p*=0.7246 | F(1, 20) = 0.84; *p*=0.3702 | F(1, 20) = 3.30; *p*=0.0841 |
| ITT | 8w/0 | F(1, 20) = 2.13; *p*=0.1595 | F(1, 20) = 0.46; *p*=0.5073 | F(1, 20) = 0.70; *p*=0.4129 |
| ITT | 8w/25 | F(1, 20) = 0.95; *p*=0.3417 | F(1, 20) = 3.80; *p*=0.0656 | F(1, 20) = 0.35; *p*=0.5609 |
| ITT | 8w/45 | F(1, 20) = 0.00; *p*=1.0000 | F(1, 20) = 1.31; *p*=0.2651 | F(1, 20) = 0.62; *p*=0.4397 |
| ITT | 8w/90 | F(1, 20) = 0.03; *p*=0.8554 | F(1, 20) = 9.20; *p*=0.0066 | F(1, 20) = 0.01; *p*=0.9377 |
| ITT | 8w/120 | F(1, 20) = 0.08; *p*=0.7825 | F(1, 20) = 0.49; *p*=0.4923 | F(1, 20) = 0.08; *p*=0.7825 |
