# Supplementary material for: Developmental Environmental Chronotoxicity by Polystyrene Nanoplastics Exacerbates High‐Fat Diet‐Induced Pediatric MAFLD Through Circadian Disruption and Mitochondrial Dysfunction
Source: Adv Sci (Weinh). 2026 Sep 27:e77840. Online ahead of print. doi: 10.1002/advs.77840 (PMC13616410; doi:10.1002/advs.77840)
Supplement: Supplementary file 2 [file ADVS-9999-e77840-s002.docx]

**Supplementary Fig. 1 | Water consumption, food intake and energy intake during the 10-week intervention period.**

**a,** Weekly water consumption normalized to body weight in juvenile (3-week-old) and adult (8-week-old) mice receiving the indicated treatments (NC, PS, HFD, and HP).

**b,** Weekly food intake normalized to body weight in juvenile and adult mice under the indicated experimental conditions.

**c,** Weekly energy intake normalized to body weight, calculated from food consumption and the corresponding caloric density of each diet.

Data are shown as mean ± SD (n = 6 mice per group). Statistical analyses were performed using two-way ANOVA followed by Tukey’s multiple-comparison test. *****p* < 0.0001; ns, not significant. NC, normal chow; PS, PS-NPs exposure; HFD, high-fat diet; HP, combined HFD and PS-NPs exposure.

**Supplementary Fig. 2 | Physicochemical characterization and hepatic distribution of GFP-labeled PS-NPs.
a,** Representative scanning electron microscopy (SEM) images showing the dispersion stability of PS-NPs suspensions monitored over three consecutive days. Scale bar, 500 nm.
**b,** Hydrodynamic diameter distribution and zeta potential profile of PS-NPs determined by dynamic light scattering (DLS) analysis.
**c,** Representative fluorescence micrographs and quantitative analysis of hepatic GFP-positive PS-NPs signals in juvenile (3-week-old) and adult (8-week-old) mice under the indicated experimental conditions. White arrows denote PS-NPs-associated fluorescence signals. Scale bars, 100 μm for low-magnification images and 20 μm for high-magnification images.
Data are presented as mean ± SD (n = 6 mice per group for fluorescence quantification). Statistical analyses were performed using two-way ANOVA followed by Tukey’s multiple-comparison test for panel c. An unpaired two-tailed Student’s t-test was used for comparisons of PS-NPs physicochemical parameters where applicable. ns, not significant. NC, normal chow; PS, PS-NPs exposure; HFD, high-fat diet; HP, combined HFD and PS-NPs exposure.

**Supplementary Fig. 3 | Age-related responses to PS-NPs and HFD exposure in body composition and glucose homeostasis.**

**a,** Changes in body weight during the experimental period, calculated as the difference between final and baseline body weights.

**b,** Adiposity index, defined as total fat mass normalized to body weight (%), in juvenile (3-week-old) and adult (8-week-old) mice under the indicated treatment conditions.

**c,** Glucose tolerance test (GTT). Blood glucose concentrations were monitored following intraperitoneal injection of glucose (1 g/kg body weight).

**d,** Insulin tolerance test (ITT). Blood glucose concentrations were measured after intraperitoneal administration of insulin (0.75 U/kg body weight). To minimize the risk of severe hypoglycemia during ITT, glucose solution (1 g/kg body weight) was prepared for emergency administration when necessary.

Data are presented as mean ± SD (n = 6 mice per group). Statistical analyses were performed using two-way ANOVA followed by Tukey’s multiple-comparison test. **p* < 0.05; ***p* < 0.01; ****p* < 0.001; *****p* < 0.0001; ns, not significant. NC, normal chow; PS, PS-NPs exposure; HFD, high-fat diet; HP, combined HFD and PS-NPs exposure.

**Supplementary Fig. 4 | SR9011 alleviates FFA and PS-NPs-induced lipid accumulation and mitochondrial impairment in hepatocytes.**
**a,** Representative Oil Red O staining images and quantitative analysis of lipid accumulation in HepG2 and Huh7 cells exposed to combined FFA and PS-NPs treatment (FP) with or without SR9011 at the indicated concentrations. Scale bars, 50 μm.
**b,** Intracellular triglyceride (TG) levels in HepG2 and Huh7 cells following the indicated treatments.
**c,** Representative JC-1 fluorescence images and quantitative analysis of mitochondrial membrane potential in HepG2 and Huh7 cells. Mitochondrial membrane potential was evaluated based on the JC-1 aggregate-to-monomer fluorescence intensity ratio. Scale bars, 100 μm.
**d,** Intracellular ATP content in HepG2 and Huh7 cells under the indicated experimental conditions.
FP represents combined treatment with FFA and PS-NPs. Data are presented as mean ± SD (n = 6 per group). Statistical comparisons were performed using one-way ANOVA followed by Tukey’s multiple-comparison test. **p* < 0.05; ***p* < 0.01; ****p* < 0.001; *****p* < 0.0001; ns, not significant.

**Supplementary Fig. 5 | Concentration-dependent effects of PS-NPs on hepatocyte viability *in vitro*.**

**a,** Cell viability of HepG2 and Huh7 cells treated with different concentrations of PS-NPs (0, 5, 20, 50, and 100 μg/mL). Cell viability was expressed relative to the untreated control group.

Data are presented as mean ± SD (n = 6 per group). Statistical differences were assessed using one-way ANOVA followed by Tukey’s multiple-comparison test. **p* < 0.05; ***p* < 0.01; ****p* < 0.001; ns, not significant.
